# A bacterial family of fatty acid acyltransferases related to the *Shigella* effector IcsB

**DOI:** 10.1128/mbio.03890-25

**Published:** 2026-03-17

**Authors:** Waad Bajunaid, Kyle Tomaro, Anwer Hasil Kottarampatel, Geneviève F. Desrochers, Mathieu Lavallée-Adam, John P. Pezacki, François-Xavier Campbell-Valois

**Affiliations:** 1 Department of Chemistry and Biomolecular Sciences and Centre for Chemical and Synthetic Biology, University of Ottawa 538781, Ottawa, Ontario, Canada; 2 Host-Microbes Interaction Laboratory, University of Ottawa 6363, Ottawa, Ontario, Canada; 3 Department of Biochemistry, Microbiology and Immunology and Centre for Infection, Immunity and Inflammation, University of Ottawa 151173, Ottawa, Ontario, Canada; 4 Department of Biochemistry, Microbiology and Immunology and Ottawa Institute of Systems Biology, University of Ottawa 175134, Ottawa, Ontario, Canada; Cornell University, Ithaca, New York, USA

**Keywords:** *Shigella*, *Burkholderia*, *Robbsia*, *Sodalis*, *Achromobacter*, *Desulfonatronospira*, *Chromobacterium*, type III secretion system, acyl transferase, fatty acid, IcsB, BopA

## Abstract

N^ε^-lysine-protein fatty acid acyltransferase (k-FAT) is a new class of enzyme found in bacteria that express either a MARTX toxin with a Rho-interacting domain (RID) or a type III secretion system (T3SS). The prototype k-FAT is the T3SS effector IcsB, which enables enteropathogenic *Shigella* species to evade destruction by autophagy in host cells. We mined protein databases using the acyltransferase domain of IcsB to expand this family. In addition to the known BopA homolog from *Burkholderia pseudomallei*, we identified several additional IcsB-like k-FATs in β- and γ-proteobacteria. These new proteins are poorly characterized and are primarily found in bacteria harboring a T3SS. Despite high sequence diversity, structure predictions indicate a highly conserved spatial disposition of catalytic residues. Furthermore, yeast cytotoxicity and in-gel acyltransferase activity depend on the integrity of the catalytic site, which supports the idea that most of the IcsB homologs identified are also acyltransferases. Finally, we used mass spectrometry to identify the yeast proteins acylated by seven of these novel enzymes. These proteins share a variety of substrates with IcsB, including membrane- and vesicle-associated proteins, such as SNAREs and small GTPases of the Rho, Rab, and Ras families. Taken together, this study provides evidence of a family of k-FAT related to IcsB.

**IMPORTANCE:**

IcsB-like k-FATs are found in the related Pseudomonadota and Thermodesulfobacteriota phyla, suggesting that they are a recent biochemical innovation. Like IcsB, new k-FATs are primarily found in proteobacterial species with a T3SS. This leaves open the possibility that they may play a role in the colonization of plants or animals. However, we characterized one k-FAT from an environmental bacterium that is unlikely to possess a T3SS. Additionally, measurable fatty acid acyltransferase activity was not detected in approximately 25% of the proteins tested. These results imply that the IcsB-like k-FAT family has undergone functional diversification and may have a more complex evolutionary origin than previously thought. In summary, this study describes the properties of the IcsB-like k-FAT family and presents yeast-based assays for characterizing new family members and unrelated proteins with similar fatty acid acyltransferase activity.

## INTRODUCTION

*Shigella* spp. cause hundreds of millions of infections and approximately 200 k deaths per year (1). To infect the colonic mucosa, *Shigella* uses a type III secretion system (T3SS) to transfer protein translocators and effectors inside host cells. This leads to the formation and rupture of a *Shigella*-containing entry vacuole that allows invasion of the cytosol (2). There, using a cell surface protein called IcsA, *Shigella* remodels the actin microfilament at one of its poles (3), thereby propelling itself into neighboring cells in a process called cell-to-cell spread. The consequence of this phenomenon is the formation of a *Shigella*-containing dissemination vacuole that, upon rupture, releases the bacterium inside the neighboring cell. Although translocators are required for the rupture of entry and dissemination vacuoles, the double membrane of the latter also requires the intervention of additional effectors (4).

IcsB is one of several protein effectors that are translocated into host cells by the T3SS to facilitate their colonization. In a seminal paper, Ogawa et al. showed that IcsB is important for escape from autophagy during cell-to-cell spread (5). Using a reporter of the T3SS activity, we showed that IcsB facilitates the escape of actively secreting *Shigella* from dissemination vacuoles labeled with the autophagosome marker LC3 (6). The mechanism by which IcsB contributes to escaping from autophagy was initially suggested to be through masking a binding site on IcsA that is recognized by the protein ATG5 (5), a key component of the autophagy pathway, thereby preventing the accumulation of LC3 around *Shigella*. An alternative model is that IcsB merely facilitates escape from the dissemination vacuole (6, 7), therefore reducing the colocalization of bacteria with the vacuole-associated LC3 (reviewed in references 2, 4).

Pioneering *in silico* analyses revealed that the central domain of IcsB, which is also found in the homologous BopA (*Burkholderia* spp.), and the more distantly related Rho-interacting domain (RID) of MARTX toxins (*Vibrio* spp.), adopts an inverted papain-like fold with a Cys-His-Asp or a Cys-His catalytic site (8). Biochemical analyses confirmed that the MARTX RID and IcsB are the founding members of two families of N^ε^ lysine (k)-protein fatty acid acyl transferases (k-FATs), which preferentially acylate small GTPases (9, 10). Stearyl, oleyl, and, to a lesser extent, palmitoyl modifications on the small GTPase RhoA were specifically identified in human cells expressing IcsB (10), suggesting that IcsB can tolerate some variation in the length and saturation of the acyl chain. In agreement with the targeting of Rho GTPases, IcsB’s acyltransferase activity was associated with the recruitment of filamentous actin around the *Shigella*-containing entry vacuole in host cells (10–12). Further investigation has also revealed that the acylation of Rab35 by IcsB facilitates *Shigella* vacuolar escape (13). This corroborates mass spectrometry data, which had previously indicated that members of the Rab GTPase family can also be acylated by IcsB (10). Additional IcsB substrates are also associated with membranes, including Ras-related GTPases, SNAREs, septins, and multivesicular body proteins (10). The latter include the charged multivesicular body protein 5 (CHMP5), a poorly characterized accessory component of the endosomal sorting complex required for transport III (ESCRT-III). Interestingly, CHMP5 is essential for the colocalization of LC3 around *Shigella* during cell-to-cell spread, and its acylation by IcsB appears to be crucial for vacuolar escape (10). Consistent with this model, the host SIRT2 lysine deacetylase can remove the fatty acid from several IcsB substrates, including CHMP5, thereby restricting *Shigella* vacuole escape (14). Notably, none of the core components of the autophagy pathway are known to be acylated by IcsB (10). Nevertheless, BopA, the only partially characterized IcsB homolog prior to this study, has also been suggested to promote the escape of *Burkholderia pseudomallei* from autophagy (15). BopA orthologs have been identified through homology searches in *B. oklahomensis*, *B. mallei,* and *B. thailandensis* (8, 16). Prior to this study, it was unclear whether other bacterial species besides *Burkholderia* and *Shigella* possessed IcsB-like acyltransferases.

Here, we have characterized 11 bacterial proteins that possess an IcsB-like acyltransferase catalytic site. Similarly to IcsB, most of these proteins can kill yeast and transfer fatty acids to a variety of membrane-associated proteins. These data allow us to significantly expand the IcsB-like k-FAT family.

## RESULTS

### Sequence similarity searches identify IcsB-like k-FATs with a conserved catalytic site

**Fig 1:**
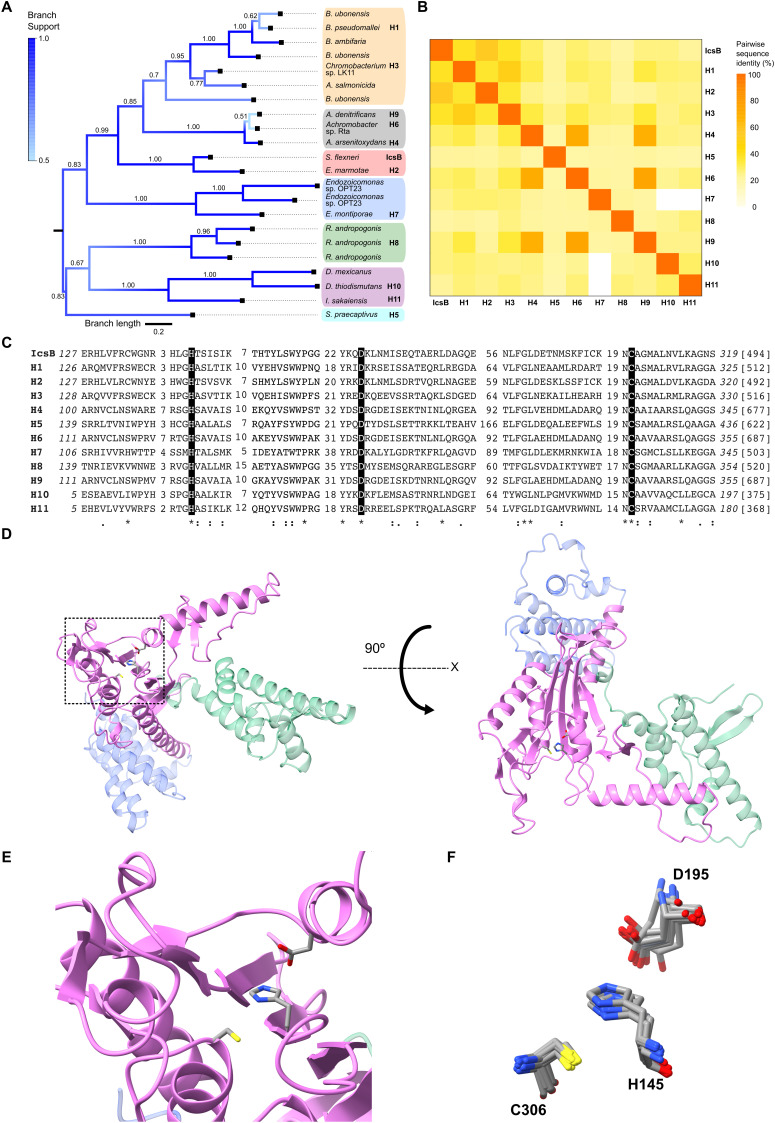
Sequence and predicted structure conservation identify a family of IcsB-like k-FATs. (**A**) Phylogenetic tree of proteins that were selected based on their sequence identity to the catalytic domain of IcsB. The previously studied BopA (H1) and representative homologous proteins (H2-H11), which are spread throughout the tree’s clades, were selected for further study. The scale bar below the tree indicates branch length. The branches are colored according to the posterior probabilities in the branch support heatmap in the top left corner. The corresponding numerical values are indicated at each branching point. Clades are colored in the following manner (Table S1): *Burkholderia* (includes *Chromobacterium* spp. and *Aeromonas salmonicida*), yellow; *Achromobacter*, gray; *Shigella* and *Escherichia*, red; *Endozoicomonas, blue; Robbsia*, green; the environmental bacteria *Desulfobotulus mexicanus*, *Desulfonatrospira thiodismutans,* and *Ideonella sakaiensis*, purple; and *Sodalis*, cyan. (**B**) Pairwise sequence identity of the catalytic domain of selected proteins. (**C**) Sequence alignment of the most conserved region of the catalytic domain of selected proteins. Stars (*) indicate fully conserved positions. Colons (:) and periods (.) indicate positions that are well preserved but have a progressively lower level of conservation. Conserved catalytic residues that aligned with histidine 145, aspartate 195, and cysteine 306 of IcsB are highlighted in black. Starting and ending residue numbers of the catalytic domain (italicized) and total sequence length (brackets) are shown. Numbers within the alignment indicate the number of residues found between two conserved regions. (**D**) Prediction of the tertiary structure of IcsB with AlphaFold3. The extended catalytic domain is in pink (residues 127–364), the N-terminal domain in green (residues 1–127), and the C-terminal domain in blue (residues 365–494). The side chains of the catalytic residues are represented as sticks in the boxed area. The structure in the right panel is rotated 90° counterclockwise to show the topology of the inverted papain-like fold of the catalytic domain. (**E**) Zoom view of the boxed area of panel D with the side chain of the catalytic residues represented as sticks. (**F**) Superimposition of the residues forming the catalytic triad in homologs H1-H11 upon alignment of their catalytic domain onto that of IcsB using structural predictions.

Since the initial report of homology between IcsB, BopA, and the RIDs of MARTX toxins (8), we have wondered whether new IcsB homologs have been added to databases. To investigate this possibility, we used PSI-BLAST to query the non-redundant protein database with the IcsB catalytic domain. This yielded approximately 1,900 sequences; the top 5% of all hits were IcsB from different *Shigella* spp. (sequence identity > 96%, E< 2 × 10^−68^). To focus on diverse high-quality sequences, we discarded grossly truncated sequences and those with greater than 75% multiple pairwise identity. The RIDs from MARTX toxins formed a distinct group with less than 20% sequence identity to IcsB (E> 3 × 10^−9^), suggesting that they constitute a separate family (17). Therefore, we discarded RIDs to focus on proteins from the IcsB family. This yielded 20 new homologs in addition to IcsB and BopA (H1). We constructed a phylogenetic tree illustrating the overall similarity of the catalytic domain across this set of proteins (Fig. 1A). The tree comprised seven clades, including proteins from β- and γ-proteobacteria, as well as two proteins from the Thermodesulfobacteriota phylum that were previously classified as δ-proteobacteria. Despite the high sequence diversity of these proteins, the posterior probabilities for each branching point indicate that the tree is reliable. The most populous clade, which is located at the top of this tree, consists mostly of *Burkholderia* spp. The exception was the nested clade comprising *Chromobacterium* sp. LK11 and *Aeromonas salmonicida*. The other clades mostly group related bacteria, providing additional support to the tree. Interestingly, *Shigella* IcsB belongs to the same clade as an *Escherichia marmotae* homolog H2. IcsB and H2 are encoded in syntenic T3SS loci of the large plasmids pINV and pEM148, which share a common ancestry (18). Notably, the IcsB catalytic triad H145-D195-C306 (8, 10) is conserved across all proteins in this tree.

Next, we selected 11 homologs (H1–H11), including the partially characterized BopA (H1), to investigate all seven tree clades (Fig. 1A; Table S1). The sequence identity of the catalytic domain of these homologs with that of IcsB ranged from 21% to 54% (Fig. 1B and C). We also generated a multiple sequence alignment with their full-length sequences (Fig. S1). Nine of the homologs have an N-terminal domain similar in size to that of IcsB. Notably, the N-terminal domain of IcsB contains a T3SS secretion signal (19). The presence of a T3SS has indeed been reported in some of the bacterial genomes that encode these novel IcsB homologs (20–22). Furthermore, the occurrence of at least one non-flagellar T3SS in eight of the 10 corresponding bacterial genomes was predicted using TXSScan software (23) (Table S2). In contrast, H10 and H11 lack an N-terminal domain equivalent to that of IcsB and are found in environmental bacteria that do not harbor a T3SS according to TXSScan. Furthermore, all the homologs but H7, H10, and H11 were predicted to be T3SS effectors by the software DeepSecE (24) (Table S3). We noticed that the sequence conservation observed in the catalytic domain extended into a contiguous C-terminal segment ending at residue 364 in IcsB (Fig. S1). We then calculated the Root Mean Square Deviation (RMSD) of this extended catalytic domain with that of IcsB (residues 128–364) using predicted tertiary structures (Fig. 1D and E; Table 1). The RMSDs for the catalytic domain ranged from 2.9 Å to 10.2 Å, while those in the better aligned regions, which were obtained by pruning unconserved loops, spanned a narrower window from 0.56 Å to 0.96 Å. The most variable region is a loop adjacent to D195. As expected, H2, the homolog with the highest sequence identity, displayed the lowest RMSD, while H7, which was among the most divergent, displayed the highest RMSD. We then aligned the predicted structure of the catalytic domain of H1-H11 onto that of IcsB. Remarkably, we observed an excellent superposition of their catalytic residues (Fig. 1F). These observations suggest that these proteins may share the same enzymatic activity despite their low sequence identity.

**TABLE 1:** Pairwise RMSD of IcsB toward its homologs according to predicted structures

Proteins	PTM^*a*^	Residue number^*b*^	Aligned residues	RMSD aligned (Å)	Aligned residues after pruning	RMSD pruned (Å)
IcsB	0.84	128–364	NA^*c*^	NA	NA	NA
H1	0.82	127–373	230	2.88	172	0.89
H2	0.81	128–366	235	1.78	214	0.56
H3	0.83	129–378	227	1.89	193	0.87
H4	0.74	101–394	237	5.54	174	0.82
H5	0.66	160–504	223	6.10	181	0.88
H6	0.73	112–404	237	6.24	166	0.82
H7	0.84	107–389	224	10.2	144	0.96
H8	0.71	141–400	215	4.43	151	0.88
H9	0.76	112–404	237	5.84	164	0.77
H10	0.85	6–239	208	4.14	152	0.83
H11	0.84	7–222	204	7.91	130	0.85

^*a*^ AlphaFold3 predicted template modeling (PTM) score for the structure of the full-length proteins.

^*b*^ This indicates the region that was used for the RMSD calculation. It corresponds to the extended catalytic domain described in Materials and Methods.

^*c*^ NA, not applicable.

### Several IcsB homologs are acyltransferases whose expression is toxic to yeast

**Fig 2:**
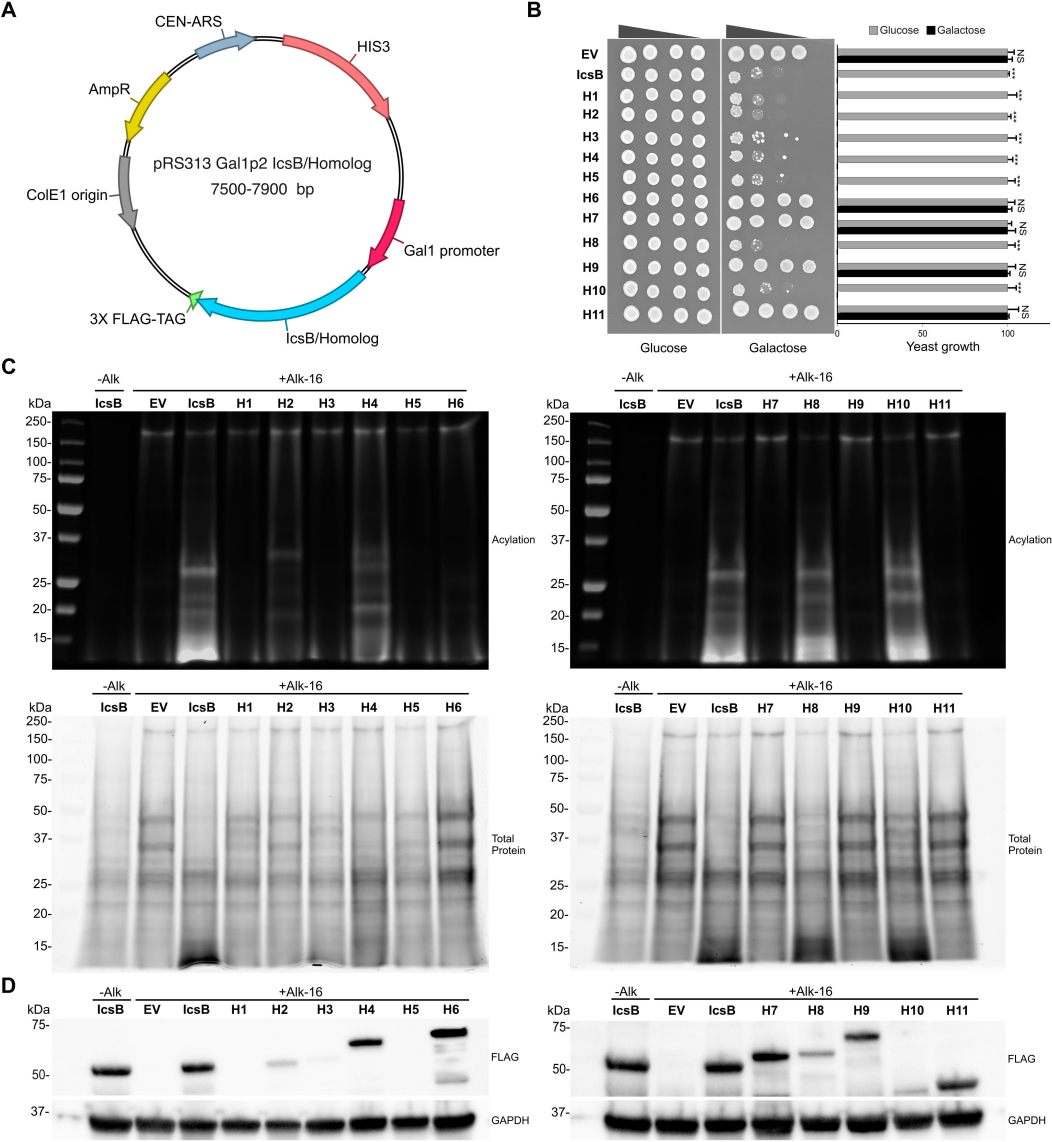
A subset of novel k-FATs exhibits yeast toxicity and IcsB-like fatty acid acyltransferase activity. (**A**) Map of the pRS313 Gal1p2 centromeric plasmid used for the galactose-inducible expression of IcsB and its homologs. (**B**)The expression of IcsB and seven of its homologs in the presence of galactose inhibits yeast growth compared to the empty vector (EV) control. The glucose plate indicates that there is no growth inhibition in the absence of expression and that similar amounts of each strain were plated. Four dilutions were plated (non-diluted, 1/10, 1/100, and 1/1,000). The graph represents the quantification of growth on three independent replicates of the 1/1,000 dilution. The error bars represent the standard deviation of the mean. A Student’s t-test with unpaired data and a 95% CI was performed (*, *P* < 0.05; **, *P* < 0.1, ***, *P* < 0.01). (**C**) Protein acylation in the membrane protein fraction of yeast expressing IcsB and its homologs according to the in-gel fluorescence acyltransferase assay (upper panels). -Alk IcsB is the IcsB sample prior to the addition of Alk-16. -Alk IcsB, Alk-16 EV, and Alk-16 IcsB were loaded on both gels to serve as controls. This acylation assay was performed using the strains from B that were induced with galactose for six hours in the presence of Alk-16 for the final 4 h. The total protein in these samples were assessed using the TGX stain (bottom panels). (**D**) The expression of IcsB and of its homologs was assessed by immunoblotting with an antibody binding to their 3× FLAG tag and with the GAPDH as a loading control.

The expression of IcsB is toxic to yeast, but point mutations in the catalytic triad rescued growth (10, 25). Therefore, we used a cytotoxicity assay to determine if IcsB homologs kill yeast as well. We established a centromeric expression plasmid system based on the GAL1 promoter, which allowed the galactose-inducible expression of full-length IcsB or its homologs with a C-terminal 3× FLAG tag (Fig. 2A). The expression of WT IcsB from this plasmid was toxic to the budding yeast *S. cerevisiae,* while that of mutants H145A and D195A was not (Fig. S2A). Surprisingly, the C306A mutant was as toxic as the WT. It was reported that the C306A mutant was well tolerated by yeast when it was coexpressed with its chaperone IpgA (10). Accordingly, we observed that coexpressing the C306A mutant with IpgA allowed growth of yeast expressing the C306A, but not the WT IcsB (Fig. S2B). For simplicity, we compared the toxicity of the WT homologs to IcsB in the absence of the chaperone. The results of this assay indicated that BopA (H1) and six out of the 10 novel homologs were toxic (Fig. 2B): H2–H5, H8, and H10. To measure the relative level of toxicity of each homolog, we quantified the pixel intensities of each spot at the 1/1,000 dilution in three independent experiments. This analysis showed that the viability of yeast expressing each homolog was reproducible and that toxic homologs were indistinguishable from one another. To determine whether these proteins had acyltransferase activity, we used a click chemistry method to detect the transfer of fatty acids to yeast proteins (10). This approach is based on a copper-catalyzed cyclization reaction between a fatty acid alkyne attached to proteins and a fluorescent azide dye, which enables the direct detection of fatty-acylated proteins on an SDS-PAGE gel. To minimize the adverse effects of these proteins’ toxicity, they were expressed for only six hours in the presence of galactose, with the last four hours also in the presence of 17-octadecynoic acid (Alk-16). An equal amount of proteins from each strain was labeled and analyzed by SDS-PAGE. To improve signal detection, the detergent-extracted proteins from the membrane fraction, resulting from the centrifugation of the zymolyase lysate, were analyzed. In the absence of Alk-16, no detectable signal was observed (Fig. 2C). In the presence of Alk-16, a band of approximately 150 kDa was observed in all conditions, including the empty vector (EV), suggesting that it corresponded to a protein that was endogenously acylated. In yeast expressing IcsB, several additional bands with molecular weights below 37 kDa appeared. A similar pattern was present in yeast expressing four out of the seven toxic proteins, H2, H4, H8, and H10, while it was absent in nontoxic homologs. The total protein analysis revealed that the extract from yeast expressing toxic proteins generally had a similar total protein content to that from yeast expressing nontoxic proteins (H6, H7, H9, and H11) or harboring the empty vector. Using immunoblotting against the 3× FLAG epitope, we routinely confirmed the expression of all but three proteins. We were never able to detect the expression of H1, while H3 and H5 were poorly expressed, rendering their detection difficult when run alongside the highly expressed homologs. This indicates that H1, H3, and H5 are expressed at much lower levels than the other homologs (Fig. 2D). Further attempts to increase the fluorescent signal, such as loading more proteins, increasing the labeling time, or increasing the concentration of Alk-16, failed to reveal the acylation activity for toxic proteins H1, H3, and H5. AlphaFold3 predicted that palmitic acid would dock in the catalytic site of seven of the 11 homologs (Table S4). Since this group contains both toxic and non-toxic homologs, the inability to bind fatty acids does not appear to cause a lack of activity in the acylation assay. We also tested myristate (Alk-12), palmitate (Alk-14), and oleate (Alk-16”) alkynes in the in-gel acylation assay (Fig. S3). Proteins that exhibited acylation activity with Alk-16 did so with these alternative fatty acids, while the activity of the remaining homologs was still undetectable. Therefore, the status of the nontoxic homologs in the IcsB-like k-FAT family is unclear. Conversely, the low expression of toxic H1, H3, and H5 may explain why their activity was not detected in this assay. Nevertheless, it is interesting to note that all proteins exhibiting detectable acylation activity were also toxic to yeast.

### The catalytic site is required for killing yeast and the acyltransferase activity of k-FATs

**Fig 3:**
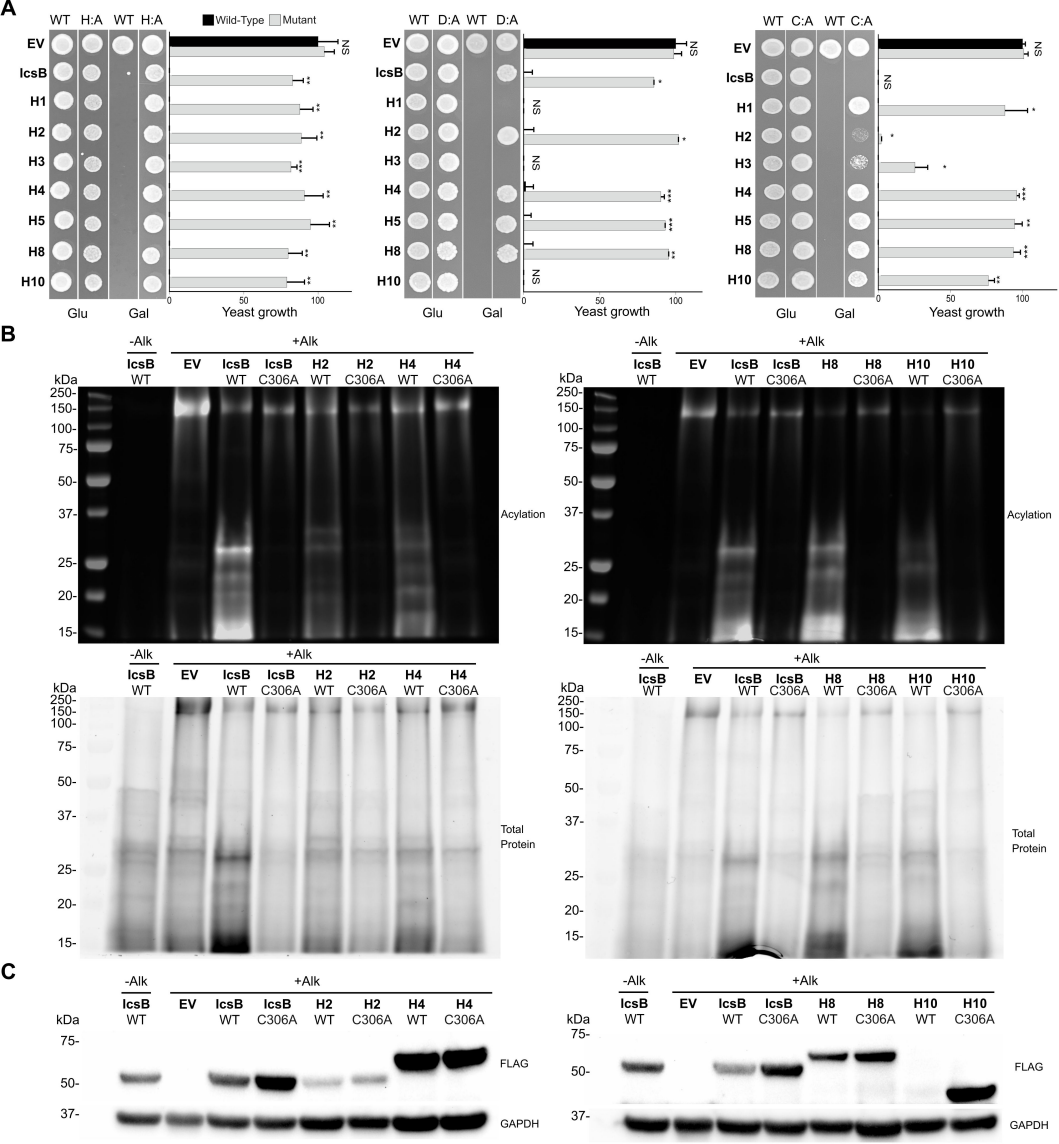
Mutations in the catalytic site of k-FATs abolish their enzymatic activity. (**A**) Yeast growth inhibition tests of catalytic residues mutants H145A (left), D195A (center), and C306A (right) compared to their WT counterpart in IcsB and in toxic homologs. The graph represents the quantification of growth on three independent replicates with the dilution of yeast cultures 1/1,000. The error bars represent the standard deviation from the mean. A Student’s *t*-test with unpaired data and a 95% CI was performed (*, *P* < 0.05; **, *P* < 0.1, ***, *P* < 0.01). (**B**) In-gel protein acylation of C306A mutants compared to their WT counterparts (upper panels). The total protein in these samples were assessed using the TGX stain (bottom panels). (**C**) The expression of IcsB and toxic homolog C306A mutants was compared to their WT counterparts by immunoblotting with an antibody binding to their 3×FLAG tag and with the GAPDH as a loading control.

Next, we investigated whether the conserved catalytic triad was necessary for inhibiting yeast growth induced by toxic homologs, as was observed with IcsB (Fig. S2). Thus, we hypothesized that a point mutation in the catalytic triad of toxic homologs would rescue yeast growth. Indeed, all H145A mutants and five out of seven C306A mutants completely rescued yeast growth (Fig. 3A). Outliers H2 and H3 C306A displayed partial rescue. As with IcsB C306A, H2, and H3 C306A may require the co-expression of their chaperone to rescue yeast growth. In contrast, the D195A mutation fully rescued growth in only four homologs. Indeed, the rescue was null for the H1, H3, and H10 D195A mutants. In summary, four homologs depend on the conserved catalytic triad to kill yeast, whereas three depend on the catalytic dyad C306 and H145. It is noteworthy that RID acyltransferase activity is mediated by a similar catalytic dyad (9). Next, we investigated whether these mutants had reduced activity in the in-gel acylation assay. Evidently, this experiment was feasible only for proteins that produced detectable signals in this assay; hence, we did not test H1, H3, and H5. Nevertheless, the IcsB, H2, H4, H8, and H10 C306A mutants were inactive (Fig. 3B). Notably, IcsB and H2 C306A exhibited no activity despite inhibiting yeast growth (Fig. 3A). This suggests that they killed yeast through a different mechanism. Rescuing IcsB C306A by coexpressing it with its chaperone, IpgA (Fig. S2), suggests that the killing mechanisms of this mutant and its closely homologous mutant, H2 C306A, may involve protein folding defects. Consistent results were obtained with H145A and D195A mutants (Fig. S4). Indeed, mutants that rescued yeast growth exhibited significantly reduced acyltransferase activity. As expected, H10 D195A, which did not rescue yeast growth, retained significant acylation activity. Overall, tseveral mutants had higher expression levels than their WT counterparts (Fig. 3C; Fig. S4). However, H8 H145A and D195A were expressed at lower levels than wild-type H8. This suggests that these mutations may destabilize the native structure of H8. Taken together, the toxicity and acylation assays on the catalytic residue mutants suggest that the toxic homologs are acyltransferases like IcsB.

### IcsB-like k-FATs acylate a wide array of yeast proteins associated with membrane transport

**Fig 4:**
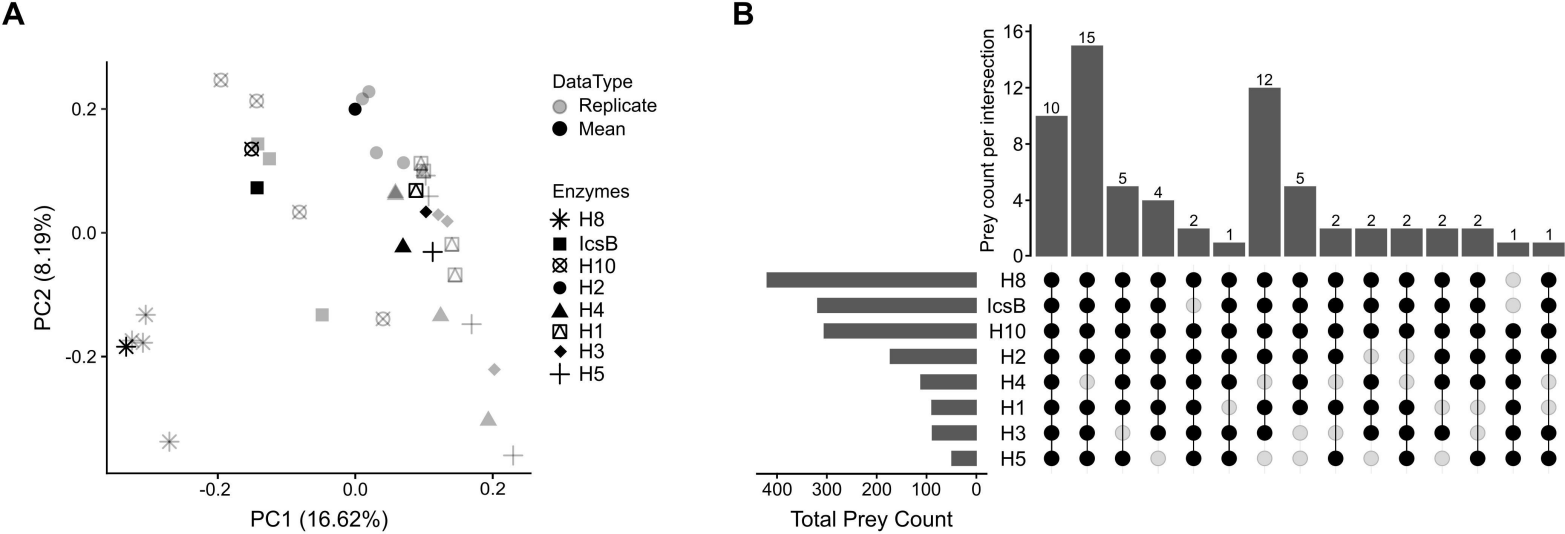
The set of proteins acylated by each k-FAT partly overlaps. (**A**) Principal component analysis of the imputed LFQ intensities. The gray shapes show each biological replicate, while the black shapes show the mean value. (**B**) The horizontal bar graph shows the total number of prey proteins with a fold change≥ 2 for each enzyme. The UpSet plot shows the intersection of the prey proteins of each acyltransferase. It includes prey proteins that exhibited a fold change≥ 2 in their LFQ intensities compared to the empty vector in at least six of the eight acyltransferases and with adjusted *P*-values ≤ 0.1 for at least one of the acyltransferases. Four biological replicates were analyzed for H1-H10 and the empty vector, while three were analyzed for IcsB.

Previous studies have shown that the biotinylation of acylated proteins with bioorthogonal labeling using a fatty acid alkyne and a biotin azide, followed by affinity purification and identification via mass spectrometry, enables the identification of a wide range of IcsB protein substrates in human cells (10). We developed a similar assay in yeast to identify proteins acylated by IcsB and its toxic homologs. Using label-free quantification (LFQ) intensities, we calculated the fold-change enrichment of the corresponding proteins in cells expressing each toxic acyltransferase versus those with an empty vector (Table S5). We performed a principal component analysis (PCA) on this data set and found that principal component 1 (PC1) and principal component 2 (PC2) accounted for 16.8% and 8.41% of the variance, respectively. This suggests that these components only captured a modest portion of the data dispersion (Fig. 4A). Biological replicates for each protein clustered reasonably well. Most of the intragroup dispersion occurred along the PC2 axis, which encompasses a smaller fraction of the variance; meanwhile, the intergroup dispersion was structured around PC1. Notably, the H8 replicates were the most tightly grouped, highlighting the consistency of H8’s behavior in this assay. H8, IcsB, and H10 yielded the highest numbers of acylated proteins (Table S5). In contrast, H1, H3, and H5, which were expressed at lower levels as discussed above, had the fewest potential substrates. Interestingly, this pattern matched with their relative positions in the PCA plot, suggesting that the number of prey proteins is a key factor influencing the dispersion of these data. We then evaluated the extent to which the acylated proteins were shared among the eight acyltransferases tested. To do so, we identified prey proteins with a fold change of at least two when comparing each acyltransferase with the empty vector and with an adjusted *P*-value smaller than or equal to 0.1 for at least one acyltransferase (Table S6). Of the 420 prey proteins associated with the homolog with the most prey proteins, H8, 173 of these were associated with a minimum of four additional acyltransferases. Similarly, 48 out of 51 prey proteins were shared in a similar way for H5, which had the fewest prey proteins. Furthermore, 66 prey proteins were associated with at least six out of the eight acyltransferases, including 10 that were identified with all the enzymes (Fig. 4B). We decided to focus on these 66 proteins to investigate the properties of proteins targeted by this acyltransferase family. First, we wondered if this set of yeast proteins is related to the set of human proteins that was previously found to be acylated by IcsB (10). To test this hypothesis, we performed an ortholog enrichment analysis with a Fisher’s exact test. This analysis indicated that the occurrence of orthologs at the intersection of both sets was higher than expected by chance (*P*= 2.6 × 10^−7^).

**Fig 5:**
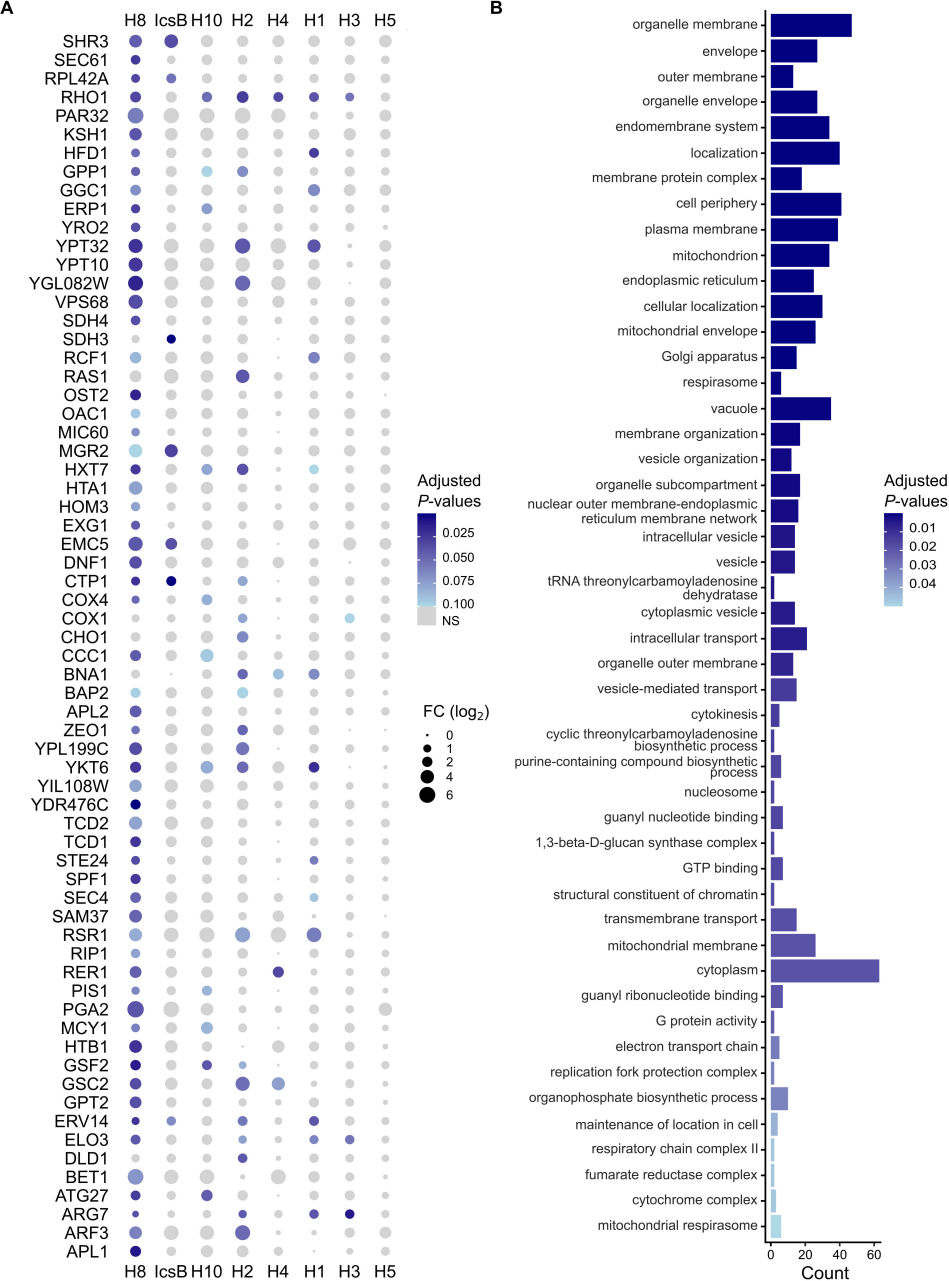
Conserved acylated proteins are associated with membrane functions. (**A**) A dot plot displaying a set of 66 proteins that are acylated by a minimum of six of the eight toxic acyltransferases. The size of the dot represents the log_2_ of the fold change of their LFQ intensities compared to the empty vector, and the color represents the adjusted FDR *P*-values as indicated in the legend. The gray circle represents data with adjusted *P*-values > 0.1. (**B**) Gene ontology term (GO-term) analyses of this set of proteins. Bars are colored according to the adjusted FDR *P*-values of each GO term.

To examine this set of conserved prey proteins in more detail, we created a dot plot to display their log₂ fold change and false discovery rate (FDR)-adjusted *P*-value (Fig. 5A). According to these parameters, the H8 data set was the most statistically robust. This finding is consistent with the low dispersion of its replicates in the PCA analysis (Fig. 4A). Thus, it served as a standard against which to validate observations in the other acyltransferases. It is noteworthy that ATG27 is the only core autophagy protein found in this data set. One of the most confidently identified shared prey proteins was RHO1, the ortholog of human RhoA, which is acylated by IcsB (10). Several other GTPases from the Rab (YPT10 and YPT32) and Ras (RAS1, SEC4, and RSR1) families, as well as SNAREs (YKT6 and BET1), were also detected. Human orthologs of RHO1, YPT32, SEC4, RSR1, and YKT6 were reported to be acylated by IcsB (10). This highlights the non-exclusive substrate preference of IcsB-like k-FATs for SNAREs and small GTPases. Furthermore, gene ontology (GO) term analysis of this conserved set of acylated proteins revealed an enrichment of terms associated with SNAREs and small GTPases (Fig. 5B), such as guanyl nucleotide binding and GTP binding (*P*-value = 0.02), organelle membranes (*P*-value = 4 × 10^−13^), intracellular transport (*P*-value = 0.005), and vesicle-mediated transport (*P*-value = 0.01). Several organelles were also identified, including the endoplasmic reticulum (*P*-value = 4 × 10^−7^), the Golgi apparatus (*P*-value = 2 × 10^−6^), and vesicles (*P*-value = 7 × 10^−5^). Notably, autophagy was not an enriched GO term (*P* = 0.70). These analyses confirmed that these acyltransferases target membrane transport, suggesting that their toxicity may stem from disrupting cellular membrane homeostasis.

## DISCUSSION

IcsB and BopA are the founding members of the IcsB-like family of N^ε^-lysine-protein fatty acid acyltransferases (IcsB-like k-FATs). Through sequence homology searches and structure prediction, we expanded the family to 20 highly diverse protein sequence prototypes, which are organized into seven clades. They mainly comprise proteins from β- and γ-proteobacteria of the Pseudomonadota phylum but also include two proteins from the related Thermodesulfobacteriota phylum. Assuming that the inhibition of yeast growth by IcsB is a general feature of the IcsB-like k-FAT family, our data validate the relationships among seven of the 11 new proteins tested. Similar to IcsB, the toxicity of these proteins to yeast was abrogated by mutating their catalytic site. We then used bioorthogonal labeling with a fatty acid alkyne, followed by in-gel fluorescence and mass spectrometry, to confirm their acyltransferase activity and yeast protein targets. Taken together, these results confirm that IcsB-like k-FATs have a broad spectrum of substrates generally associated with cellular membranes, particularly small GTPases and SNAREs.

The phylogenetic tree presented here was constructed to illustrate sequence diversity by focusing on proteins with less than 75% pairwise sequence identity. Six of the seven clades include bacteria that have demonstrated or suspected abilities to establish commensal or pathogenic relationships with animal or plant cells. These clades encompass IcsB and H1–H9. The two most studied clades include k-FATs from the pathogens *Burkholderia pseudomallei* and *Shigella* spp., which both use their T3SS to invade human cells (10, 15). *E. marmotae*, which possesses an invasion plasmid-encoded T3SS related to that of *Shigella* (18), has been identified in clinical samples (26). The other *Burkholderia* spp. and the *Chromobacterium* sp. that we have identified are not strongly associated with animal or plant infections. For instance, *Burkholderia thailandensi*s, considered a plant saprophyte, rarely causes disease (27, 28). *B. ubonensis* and *B. ambifaria* are classified in the *Burkholderia cepacia* complex, which is associated with opportunistic lung infections. Finally, this clade also includes *Aeromonas salmonicida*, whose T3SS plays a key role in the infection of freshwater fishes (29). The *Achromobacter* clade comprises bacteria that have been isolated from soil and insects. *A. arsenitoxydans* was found in soil from a pig farm (21), suggesting a possible association with this mammal. IcsB-like k-FATs are also found in *A. spanius* and the recently sequenced *A. xylosoxidans* str. JCM 9787. Representatives of these species have been associated with opportunistic human infections (30–33). The *Endozoicomonas* clade comprises poorly characterized bacteria that colonize cnidarians, such as anemones and corals (34–36). The *Robbsia* clade includes three proteins from *R. andropogonis*. This bacterium is a plant pathogen that infects maize, sorghum, coffee, and legumes (37). The *Sodalis* clade is sparsely populated with a few highly similar sequences associated with *S. praecaptivus*, a free-living bacterium that has also been linked to commensalism in insects and rare opportunistic infections in humans (38, 39). Several other *Sodalis* spp. are obligate insect commensals (40, 41); however, we did not find IcsB-like k-FATs in them. By contrast, the environmental clade includes H10 and H11, which are from bacteria that are unlikely to colonize eukaryotes.

Several T3SS effectors are toxic to the budding yeast *S. cerevisiae* (42). This phenotype has played a key role in elucidating the enzymatic activity of IcsB (10). We have demonstrated that this phenomenon is a characteristic of this enzyme family. Indeed, most family members tested share the ability to inhibit yeast growth. This is likely due to their targeting of essential proteins involved in vesicle transport and organelle maintenance, both of which are critical to cellular homeostasis. Additionally, the low endogenous protein acylation observed in *S. cerevisiae* makes this organism a suitable model for studying long-chain fatty acid acyltransferases. Indeed, we found interpreting the yeast in-gel fluorescence assay to be more straightforward than previous attempts with human tissue culture cells (10). Bioorthogonal labeling with fatty acid alkynes was used to elucidate the acyltransferase activity exhibited by four of the seven toxic homologs. These enzymes transferred the following fatty acid alkynes into target proteins: 14:0 (myristate, Alk-12), 16:0 (palmitate, Alk-14), 18:0 (stearate, Alk-16), and, to a lesser extent, 18:1 *cis* △9 (oleate, Alk-16”). It is unclear whether these differences are due to the enzymes’ substrate preferences or the bioavailability of these fatty acids. Nevertheless, stearate yielded the best signal-to-noise ratio compared to the empty vector. This may be because fewer proteins are naturally stearylated than are myristoylated or palmitoylated. Next, we used biotin azide and bioorthogonal labeling to biotinylate proteins that were stearylated by the k-FATs, which enabled their affinity purification and identification by mass spectrometry. This technique’s sensitivity allowed us to detect protein substrates of all toxic homologs, even those whose acyltransferase activity was undetectable by in-gel fluorescence. The cytotoxicity of these enzymes can complicate the identification of their protein substrates in yeast. Nevertheless, using the galactose-inducible expression system, we found that 6 h of incubation with the fatty acid alkyne optimized the balance between the yield of acylated proteins and yeast fitness. Despite these optimizations, the adjusted *P*-values for several substrates exceeded the significance cutoff (adjusted *P*-value > 0.1). Fortunately, the data for the protein H8 were statistically robust and abundant in prey proteins. Thus, the substantial substrate overlap between H8 and the other enzymes contributed to validating our data set. This finding suggests that H8 could be included as a benchmark in future studies of this k-FAT family. In addition, the great evolutionary distance between yeast and the natural plant or animal host of bacteria harboring k-FATs may introduce discrepancies. For instance, the human protein CHMP5 is a primary target of IcsB during *Shigella* infection (10). However, its yeast ortholog VPS60 has poor sequence identity, which may explain why it was not identified in our mass spectrometry experiments.

Despite their sequence and structural similarities, four homologs—H6, H7, H9, and H11 —did not exhibit any IcsB-like k-FAT features in yeast. This is especially surprising for H6 and H9, whose catalytic domains are 70% identical to that of their active clade neighbor, H4. Higher sequence divergence in other regions may affect their enzymatic activity. H7 was the only protein in our data set with an N-terminal domain that was not predicted to be a T3SS effector. Interestingly, DeepSecE analyses predicted that H7 and its two closest homologs are secreted by the T4SS. Therefore, the association of the *Endozoicomonas* clade with the IcsB-like k-FAT family is not well supported. H11 and its clade neighbor H10 are the only characterized homologs found in environmental bacteria lacking a T3SS, according to computer predictions (23). Unlike H11, however, H10 exhibited acyltransferase activity. This suggests that IcsB-like k-FATs can be found in proteins unrelated to the T3SS. Currently, their function remains unclear. On the other hand, the lack of detectable acylation activity of H6, H7, H9, and H11 suggests that they may have a more restricted range of fatty acid or protein substrates. Alternatively, their acylation activity may be regulated in a different way. For example, post-translational modifications may be necessary for activation. They may also have evolved new enzymatic activities. In this regard, the flexibility of the catalytic site in this family is highlighted by the finding that H3 and H10, unlike other toxic homologs, did not require D195 to kill yeast and that H10 D195A displayed strong in-gel acylation activity. So why is D195 conserved in H3 and H10? Although D195 is unnecessary for H3 and H10 activity in yeast, it could still be important for efficiently acylating their natural substrates. Furthermore, the Cys-His-Asp catalytic site and the predicted papain-like fold of the IcsB catalytic domain are also compatible with protease activity (8). Thus, IcsB homologs could possess this alternative enzymatic activity. Finally, we have not performed a comparative analysis of the shape of the catalytic pocket. Such an analysis may reveal features that underpin the activity of these proteins.

Notably, none of the human proteins acylated by IcsB are core components of the autophagy pathway (10). While the acylation of human CHMP5 by IcsB seems to be enough for *Shigella* to evade LC3-positive vacuoles, the purpose of IcsB-mediated acylation of SNAREs and small GTPases involved in membrane transport during infection is unclear (10, 13). Among the conserved targets of the novel k-FATs, the protein ATG27 is the only core autophagy component (43). However, the scope of this finding is limited because ATG27 is a fungal protein with no known animal or plant counterpart. On the other hand, we identified several yeast small GTPases and SNAREs that are acylated by k-FATs. The GO term analysis revealed that these enzymes primarily target membrane transport. Thus, we conclude that targeting endomembrane trafficking and homeostasis rather than autophagy itself is a hallmark of this family. This could help a bacterium harboring one of these novel k-FATs escape its vacuole, as is the case with IcsB in *Shigella* (5, 6, 10). Alternatively, it could contribute to the acquisition of nutrients or the establishment of a replicative niche in endomembranes, on the surface of host cells, or in their vicinity. Evidently, the role of the new T3SS-associated acyltransferases in the colonization of animals or plants remains to be tested. Those found in *Robbsia andropogonis* and *Sodalis praecaptivus* are of particular interest because these bacteria have been found in several plants and insects (22, 39). The uncharacterized homologs revealed here, as well as those yet to be discovered, represent an interesting area for future research on this intriguing family of enzymes.

## MATERIALS AND METHODS

### IcsB homolog sequence alignment and phylogenetic tree

The catalytic domain of IcsB from *Shigella flexneri* str. M90T (WP_031192845.1) corresponding to residues 127–319 (8) was queried against the NCBI non-redundant protein database (November 2019) using four rounds of PSI-BLAST (44). We discarded the abundant MARTX toxins, trimmed the alignment of short sequences, and removed redundant sequences to keep a subset of sequences with less than 75% multiple pairwise sequence identity. Additional BLAST searches (October 2025) were performed with the initial hits. This led to the identification of a new homolog with less than 75% multiple pairwise sequence identity and established the presence of homologs with greater than 75% multiple pairwise sequence identity in other *Burkholderia* spp. and *Achromobacter* spp. (Table S1). A phylogenetic tree was constructed with IcsB, BopA, and 20 new homologs using Phylogeny.fr (45). Several settings were tested until a tree was obtained in which the homologs found in related bacterial species were grouped in the same clade. The final settings used were sequence alignment with T-Coffee (46), alignment curing by removing positions with gaps, and tree construction over 100,000 generations with the Bayesian phylogeny algorithm MrBayes (47) using the WAG+G substitution model, which was determined to be optimal for this protein set using ProtTest3 (48). Default settings were used for the other parameters. The tree representation was generated with Figtree (49). The statistical confidence of each internal branch was evaluated using posterior probabilities. The full-length sequence alignment was performed with T-Coffee and analyzed with Clustal W.

### Structure prediction and root mean square deviation

AlphaFold3 was used to predict the structure of the full-length primary structure of IcsB and homologs H1–H11 alone or with palmitic acid (50). Next, ChimeraX was used to calculate the pairwise RMSD of the predicted structures of the apo homologs relative to that of IcsB (51). The calculation was performed for the extended catalytic domain spanning residues: IcsB, 128–365; H1, 127–373, H2, 128–366; H3, 129–378; H4, 101–394; H5 160–504; H6, 112–404; H7, 107–389; H8, 141–400; H9, 112–404; H10, 6–239; and H11, 7–222. Protein structural representations were also obtained with ChimeraX.

### *In silico* prediction of the T3SS

The presence of the non-flagellar T3SS system in bacterial species encoding BopA and the 10 novel homologs was performed using TXSScan (23) in Galaxy Pasteur (52). These analyses were performed with chromosome or virulence plasmid sequences that were obtained from the NCBI or ENSEMBL bacteria database. DeepSeqE was used to predict if the IcsB homologs were T3SS effectors (24). It was run according to the instructions provided on GitHub and with the default weights of the DeepSecE model.

### Plasmids

pRS313 and pRS316 yeast centromeric shuttle vectors (U03439.1 and U03442.1 in GenBank) were a kind gift from Cammie F. Lesser (25). These plasmids were used as templates to generate the entry expression vector pRS313/316-Gal1p1 and pRS313/316-Gal1p2. First, a synthesized fragment named GAL1p (Integrated DNA Technologies, IDT), including M13 forward and M13 reverse sequences, a multi-cloning site, and a Myc-tag immediately followed by stop codons in each reading frame, was sub-cloned by ligation of XhoI- and XbaI-generated fragments into both plasmids. Second, the M13 forward and reverse sequences were replaced with SP6 forward and BGH reverse sequences by mutagenesis PCR, yielding pRS313/316-Gal1p1. Finally, the Myc-tag was replaced with a 3× FLAG tag by mutagenesis PCR as well, yielding pRS313/316-Gal1p2. The coding sequence of IpgA (pWR100, str. M90T) was amplified by PCR from a plasmid harboring the IpgA-TEM1 coding sequence (53) and subcloned into pRS316-Gal1p1 by ligation through BamHI and EcoRI sticky ends, yielding pRS316-Gal1p1 IpgA. The coding sequence of IcsB (pWR100, str. M90T) was ligated into pRS313-Gal1p2 through BamHI and EcoRI sticky ends. The primary structure of IcsB homologs retrieved from GenBank (H1 [BopA]: WP_004528810; H2: WP_038355842.1; H3: WP_048412814.1; H4: EHK65445.1; H5: AHF79295.1; H6: WP_043548505.1; H7: WP_034879418.1; H8: WP_024901980.1; H9: WP_088447978.1; H10: WP_008870026.1; and H11: WP_054022033.1) were used for gene synthesis with *S. cerevisiae* codon usage (Bio-Basic, Markham, Ontario, except H1, which was synthesized by Biomatik, Kitchener, Ontario). Subcloning into pRS313-Gal1p2 was performed as for IcsB. The plasmids and the oligonucleotides are listed in the supplemental material (Tables S7 and S8). Point mutations at codons corresponding to conserved catalytic residues in IcsB or its homologs were introduced by mutagenesis PCR. Oligonucleotides were purchased from IDT. Phusion high-fidelity DNA polymerase, restriction enzymes, and T4 DNA ligase were from Thermo Fisher. Ligation reactions were transformed into either DH10B or DH5alpha *E. coli* strains. Sequences were verified by Sanger sequencing (Génome Québec). These plasmids are available from Addgene: pRS313 Gal1p2 (#242948), pRS313 Gal1p2 IcsB (#242949), pRS313 Gal1p2 H8 (#242950), pRS316 Gal1p1 (#242951), and pRS316 Gal1p1 IpgA (#242952).

### Yeast cytotoxicity assay

The yeast toxicity assay for IcsB and homologs was performed with the *S. cerevisiae* haploid mat α strain BY4741 (gift from CF Lesser), an auxotrophic derivative of S288C. To perform the rescue of the C306A mutant of IcsB, the pRS416 Gal1p1 IpgA was transformed into the corresponding haploid mat α strain BY4742 (gift from CF Lesser). These strains were transformed with the lithium acetate/PEG4000 protocol. In brief, one colony was picked from a freshly made plate and transferred to 4 mL of YPD broth and incubated overnight at 30°C with shaking (250 rpm). The next morning, the cells were diluted to an OD_600_ of 0.2 in 50 mL of YPD and incubated with shaking for 3 h at 30°C. Cells were then pelleted by centrifugation at 1,900 × *g* for 5 min and successively washed in phosphate-buffered saline (PBS) (Wisent, 311-425 CL) and 1 mL of 0.1 M lithium acetate (LiAc; Sigma-Aldrich, L6883), then finally resuspended in 500 µL of 0.1 M LiAc. For each transformation, 50 µL of this LiAc cell suspension was aliquoted into a microcentrifuge tube. Using a vortex, cells were homogeneously resuspended in the transformation mix (240 µL of PEG 3350 50% (wt/vol), 36 µL of 1 M LiAc, 50 µL of salmon sperm DNA, 33 µL of sterile water, and 1 µL of plasmid DNA [250–500 ng]). The cell suspension was incubated for 20–60 min at 42°C. Cells were pelleted by centrifuging for 30 s at 9,200 × *g* and washed carefully with PBS. Finally, cells were resuspended in 200 µL of PBS and plated on Petri dishes containing the relevant synthetic minimal media (SD) prepared with dropout supplements (Sigma-Aldrich, Y1751 and Y1501; USBiological Life Sciences, D9537-07). Diploid strains with pRS413 Gal1p2 IcsB and pRS416 Gal1p1 IpgA and related control strains were obtained by mating the haploid strains. Glycerol stocks of transformants in YPD broth with 25% glycerol (wt/vol) were prepared and stored at –80°C. Two days before the experiments, yeast strains were grown on the relevant SD plates. A colony was picked and incubated overnight at 30°C in SD broth with 2% raffinose to prime cells for the induction. The next day, cells were pelleted, resuspended in 100 µL NaCl 0.9% (wt/vol), and three 10-fold serial dilutions were prepared (1/10, 1/100, and 1/1,000). After that, 3–5 µL of each dilution was spotted on plates containing the relevant SD medium containing 2% glucose (wt/vol) or 2% galactose (wt/vol). Plates were then incubated at 30°C for 3 days, at which time a photograph of yeast spots was taken. The growth inhibition on galactose was measured by performing densitometry on these images with the software Image J, version FIJI (54). KaleidaGraph 4.5.4 (Synergy Software) was used to plot the data and perform the relevant statistical tests.

### Immunoblotting of yeast extracts

Fresh colonies were picked from the SD plate to inoculate the corresponding SD broth. The resulting overnight cultures were diluted to OD_600_~ 1.0 in SD medium containing 2% raffinose and incubated for 3 h at 30°C with shaking. Then, 2% galactose was added to induce protein expression, and incubation was continued under the same conditions as above for 4 h. Next, a sodium hydroxide protocol was used to lyse yeast cells (55). Briefly, 500 µL of cells were pelleted and resuspended in 100 µL of deionized water. This cellular suspension was then mixed gently with 100 µL of 0.2 M NaOH and incubated for 5 min at room temperature. Cells were pelleted and resuspended in 50 µL of 1× Laemmli containing 1 mM PMSF and incubated for 5 min in boiling water. This cell lysate was pelleted, and the resulting supernatant was transferred to a new microfuge tube. Samples were loaded on a 12% SDS-PAGE gel and transferred onto a PVDF membrane. Primary antibodies used were mouse FLAG-tag (Sigma-Aldrich, F3165), mouse Myc-tag (Genescript, A00704), and rabbit GAPDH (Bioss antibodies, BS-8789R) diluted 1/5,000, 1/1,000, and 1/10,000, respectively. Secondary antibodies used were goat anti-mouse IgG (Jackson Immunoresearch Laboratories Inc., 115-035-003) and goat anti-rabbit IgG (Jackson Immunoresearch Laboratories Inc., 111-035-003) conjugated with horseradish peroxidase both diluted 1/20,000. The membrane was visualized using a ChemiDoc XRS+ gel documentation system (Bio-Rad). HRP activity was detected using the Clarity Western ECL Substrate (Bio-Rad, 1705061), as indicated by the supplier.

### In-gel fluorescence acylation assay

Yeast strains harboring IcsB and its homologs were streaked on plates from glycerol stock. A colony was used to inoculate 4 mL of SD broth with 2% raffinose and incubated overnight with shaking at 250 rpm and 30°C. Further incubation steps described below were performed in the same conditions unless mentioned otherwise. Next morning, the cells were diluted to OD_600_~ 1 (e.g., 1:8) in 4 mL of SD broth with 2% raffinose and incubated for 3 h. Then, 2% galactose was added to the medium. The cells were incubated for two hours to induce expression. Then, 10 μM of fatty acid alkyne was added (Cayman Chemical, 17-octadecynoic acid, also known as Alk-16 [#90270], myristic acid [#13267], palmitic acid [#13266], or oleic acid [#9002078]). Cells were incubated for 4 h and collected by centrifugation at 12,000 × *g* for 5 min at 4°C. The resulting cell pellet was immediately frozen on dry ice and stored at −80°C. Typically, the next day, the cells were resuspended in 300 μL of PBS supplemented with 5 µL of zymolyase (25 units). The resuspension was incubated at 37°C for 15–20 min. If deemed necessary upon observation under the microscope, the incubation can be extended by 5-min intervals to increase spheroplast formation. Glass beads with diameters of 425–600 µm (Sigma-Aldrich, G8772), representing approximately a quarter of the resuspension volume, were added to the cells. The cell-bead slurry was then mixed 5 × 1 min using a vortex at room temperature; cells were incubated on ice for 1 min between each pulse. The insoluble fraction was isolated by centrifugation at 17,000 × *g* for 20 min at 4°C and resuspended in PBS 1% Triton X-100 supplemented with 1× cOmplete EDTA-free Protease Inhibitor (Roche, 05892791001). Proteins were extracted using end-to-end rotation for 20 min at 4°C. The proteins in the detergent phase were separated from the insoluble cell debris by centrifugation at 15,500 × *g* for 20 min at 4°C; 70 µg of proteins was reacted with rhodamine azide by copper-catalyzed azide-alkyne cycloaddition (CuAAC) (56). To do this, the protein sample was adjusted to 70 μL (e.g., 1 µg/µL) in PBS, and 100 µL of the click mix was added. The click mix was obtained by mixing these reagents in the following order: (i) 20 μL of freshly prepared 50 mM TCEP; (ii) 20 μL of 50 mM CuSO_4_; (iii) 1 µL of 100 mM TBTA; (iv) 1 μL of 100 mM rhodamine azide; and (v) the reaction volume was finally completed to 1 mL using PBS. Thereafter, the samples were protected from light and incubated at room temperature for 60 min. Then, 1 mL of acetone was added to each sample, which was then incubated at −80°C for 15 min to overnight. The proteins were recovered by centrifugation at 15,500 × *g* for 20 min at 4°C. The pellet was air-dried at room temperature and resuspended by vortexing in 25 µL 2× gel loading buffer. The samples were incubated at 95°C for 10 min and analyzed using 12% FastCast acrylamide SDS-PAGE (Bio-Rad 1610185) or 4%–15% gradient precast TGX stain-free gel (Bio-Rad, 4568083).

### Preparation of the protein samples for mass spectrometry

Yeast strains expressing the IcsB-like k-FATs were grown as described in the previous section in 40 mL of SD medium. Proteins from the insoluble fractions were extracted and conjugated with biotin azide using click chemistry. First, each sample was split into 2 × 500 µL. The following reagents were successively mixed into each sample by vortexing (i) 10 µL of 5 mM biotin azide (Sigma-Aldrich, QBD10825); (ii) 10 µL of freshly prepared 50 mM TCEP; (iii) 10 µL of 5 mM TBTA diluted 1:4 in DMSO:t-butanol; (iv) 10 µL of 50 mM CuSO_4_*5H_2_O (Sigma-Aldrich, 939315). The resulting reaction tubes were next rotated at room temperature (RT) for 2 h. Then, the unreacted click reagents were removed as follows: (i) duplicates of click reactions were pooled and incubated for 15 min to overnight with five volumes of −20°C acetone; (ii) samples were centrifuged at 17,000 × *g* for 15 min at 4°C; (iii) acetone was decanted and 750 μL of −20°C methanol was added; (iv) samples were sonicated (e.g., 5 pulses at 30% power) to break up the pellet; (v) samples were centrifuged 6,500 × *g* for 5 min at 4°C to pellet protein, and then, the supernatant was removed; (vi) steps iii to v were repeated two more times; and (vii) 650 μL of 2.5% SDS in PBS was added to protein pellets and they were sonicated (15 pulses at 30% power). Samples were either stored at −20°C until the next day or heated for 5 min at 60°C, centrifuged at 6,500 × *g* for 4 min at RT*,* and the supernatant was then transferred to a 15 mL conical. The pellet should be less than 20% of the starting material. If larger, the heating is repeated for 2–5 min at 90°C. The volume was brought to 3.5 mL in PBS and stored overnight at −20°C. The next day, 100 μL of 50% streptavidin-agarose bead slurry (Thermo Scientific, 20353) was washed 3× with 700 μL PBS in a biospin column (Bio-Rad, 7326204) at 1,000 × *g* for 1 min. Beads were then transferred to protein samples using 500 μL PBS and rotated for 180 min at room temperature. Beads were centrifuged for 2 min at 1,400 × *g* and RT; the supernatant was removed, leaving approximately 500 µL of the supernatant to facilitate the transfer of bead-attached sample into a biospin column. Samples were then washed 3× with 1% SDS, followed by washing 3× with fresh 6 M urea (Bio-Rad, 161-0731). Samples were then washed with 1× PBS, followed by washing 5× with 50 mM ammonium bicarbonate (ABC) (Thermo Scientific, 393212500). Beads were then transferred to microcentrifuge tubes using 2× 350 µL of ABC buffer. Beads were then pelleted at 1,400 × g for 2 min, and the resulting supernatant was removed; 500 µL of 10 mM DTT (Thermo Scientific, R0861) in ABC was added and heated at 65°C for 15 min. Then, 25 µL of 500 mM iodoacetamide (Thermo Scientific, 122270050) was added, and samples were rotated in the dark for 30 min, followed by centrifugation at 1,400 × *g* for 2 min and supernatant removal. Beads were then washed 3× with 500 µL of 50 mM TEAB (Thermo Scientific, 90114) buffer and stored in 100 µL TEAB buffer at 4°C until adding trypsin (Promega, V5113); 2 μL of 0.5 mg/mL trypsin was added and rotated at 37°C overnight. Beads were pelleted the next day, and the supernatant was transferred into a clean biospin column, centrifuged at 1,000 × *g* for 1 min at RT. The flow-through containing the digested peptides was collected and subjected to vacuum centrifugation at room temperature for 30 min or until completely dry.

### Mass spectrometry samples run

The samples were analyzed at the John L. Holmes Mass Spectrometry Facility (Faculty of Science, University of Ottawa), as previously described (57). Briefly, the tryptic peptides obtained in the previous section were analyzed by an Orbitrap Fusion mass spectrometer (Thermo Scientific) coupled to a Dionex UltiMate 3000 RSLCnano system (Thermo Scientific) with an in-house packed 70 µm ID × 150 mm length separation column (Polymicro Technology), Luna C18(2), 3 µm and 100 Å (Phenomenex). Samples were injected and separated through a gradient solution of 0.1% formic acid and acetonitrile in deionized water. They were applied to the column for a total of 105 min at a flow rate of 0.30 µL/min, which was broken down as follows. The peptides were separated with 2% acetonitrile for the first 7 min, then the acetonitrile gradient was linearly increased to 38% for the next 70 min, then from 38 to 98% for 5 min, remained at 98% for 10 min, then gradually decreased from 98 to 2% acetonitrile for 3 min, and finally washed with 2% acetonitrile for 10 min. Peptides were ionized using nano-ESI at an ion source temperature of 250°C with an ion spray voltage of 2.1 kV. Survey scans between 300 and 2,000 *m*/*z* were acquired at 60K resolution, and precursors with charge states +2 to +7 were filtered according to the monoisotopic precursor selection. The dynamic exclusion time was set to 30 s with a tolerance of 10 ppm. Automatic gain control settings were set to 5 × 10^5^ for full FTMS scans and 1 × 10^4^ for MS/MS scans. Precursors were isolated using a 2 m/z isolation frame, and collision-induced dissociation (CID) fragmentation was performed with a collision energy of 35%.

### Mass spectrometry data analyses

Analyses of the raw mass spectrometry data were performed as previously reported (57). In brief, the data were processed using the Trans-Proteomic Pipeline (TPP v6.3.0 Arcus, build 202305021110-8944 for WindowsNT-x8664) (58), as follows. The raw files were converted to mzML using ProteoWizard’s msconvert (version 3.0.22340) (59). Comet (release 2023.01 rev.2) (60) was used for peptide-spectrum matching against a protein sequence database of *Saccharomyces cerevisiae* strain ATCC 204508/S288C (Proteome ID, UP000002311) from the UniProtKB database along with the ‘common contaminants’ from the Max Planck Institute of Biochemistry (61), and DECOY-prefixed reverse protein sequences, therefore creating a total protein library of 12,610 sequences. Semi-tryptic peptides with at most three miscleavages were searched with a precursor mass tolerance of 20 ppm. Carbamidomethylation (C) was considered a static modification, and oxidation (M), deamidation (NQ), and acetylation (n) were considered variable modifications. Fragment bin tolerance was set to 1.0005 for the ion trap ms/ms and up to triple-charged ions were allowed. The confidence of the Comet peptide-spectrum matches was assessed using PeptideProphet (62), and protein identification confidence evaluation was performed with ProteinProphet and iProphet (63, 64). Protein identification was deemed confident if they showed a False Discovery Rate (FDR)< 1%. Label-free quantification was performed using StPeter (version 1.4.0) (65). Protein sample size was set at 20 µg, and degenerate peptide quantification was allowed. Grubb’s tests were used to identify potential outliers in the biological replicates of each set. On this statistical basis, one of the replicates of IcsB was discarded (*P*-value = 0.05). Therefore, three biological replicates were used for further analysis of IcsB, while four biological replicates were used for the empty vector control and the new acyltransferases. Next, proteins detected in a minimum of one replicate (LFQ > 0), but undetected (LFQ = 0) in one or many of the remaining replicates for a given sample type, were imputed using randomly selected values picked from the lowest 10% of all LFQ intensities with uniform probabilities in the corresponding data set. The PCA analysis was performed on the imputed data set of each replicate using the R function prcomp(data, center = TRUE, scale= TRUE) using the Stats package (version 4.4.1). The function autoplot (pca, data) was used to generate the initial PCA plot, which was then customized with ggplot2 (version 3.5.2); the UpSet plot was generated using the ComplexUpset package (version 1.1.3), and the dot plot with ggplot2. The fold change in abundance (FC) was calculated with the imputed LFQ data set as follows: FC = LFQ bait/LFQ empty vector control.

*P*-values were calculated for each bait-empty vector pair using a one-tailed greater alternative unpaired *t*-test with unequal variance. These were adjusted for multiple hypothesis testing using the Benjamini & Hochberg FDR method. They were then merged into a single data set, and all missing FDR-adjusted *P*-values from proteins that were absent in individual bait data sets were set to 1. The data set was filtered to select for further analyses the proteins with a fold change of ≥ 2 and an FDR-adjusted *P*-value of ≤ 0.1 in at least one of the baits. The R program (version 4.4.1 ran on R Studio) was used to generate the figures. The ortholog enrichment analysis was performed by comparing the data set of human proteins acylated by IcsB (10) to the yeast proteins acylated by at least six of the eight IcsB-like k-FATs (Fig. 5A). The yeast proteins were mapped to all their UniProtKB/Swiss-Prot identifiers with their gene names using UniProt’s identifier mapping service and API (66–68). The Orthologous Matrix Browser (OMA) (69) was used to obtain the list of human-yeast orthologs. The statistical significance of ortholog enrichment was obtained using a one-tailed Fisher’s exact test, which was computed using the R function dhyper (x, m, n, k), where n represents the number of human proteins acylated by IcsB with yeast orthologs, m represents the total number of human proteins with yeast orthologs that are not included in “n,” k represents the number of acylated yeast proteins with human orthologs, and x represents the number of proteins common to “k” and “n.” This test returned the *P*-value for the number of yeast proteins that are acylated by at least six of the eight IcsB-like k-FATs and that have orthologs among the human proteins that were previously reported to undergo acylation by IcsB (10). The gene ontology (GO) analysis was done with Ontologizer 2.1 (Build: 20160628-1269) using the GO data set version 1.2 released on 2023-10-09 mapped to the *S. cerevisiae* genome with PANTHER version 17.1, where the population was the proteome of *S. cerevisiae,* and the study set included the list of proteins present in at least six of the eight homologs. Finally, the GO-term plot was created with ggplot2 in R. 

## Data Availability

The mass spectrometry data are available from the Mass Spectrometry Interactive Virtual Environment (MassIVE) at MSV000098053.

## SUPPLEMENTAL MATERIAL

Supplemental material mbio.03890-25-s0001.pdf Supplemental figures and tables.

Table S5 mbio.03890-25-s0002.xlsx Mass spectrometry raw data, fold change enrichment, and statistical analyses of proteins retrieved from IcsB and toxic homolog samples

Table S6 mbio.03890-25-s0003.xlsx Acylated proteins based on the mass spectrometry data used in the UpSet analyses.

ASM does not own the copyrights to Supplemental Material that may be linked to, or accessed through, an article. The authors have granted ASM a non-exclusive, world-wide license to publish the Supplemental Material files. Please contact the corresponding author directly for reuse.
