# Supplementary material for: A bacterial family of fatty acid acyltransferases related to the Shigella effector IcsB
Source: mBio. 2026 Mar 17;17(4):e03890-25. doi: 10.1128/mbio.03890-25 (PMC13059803; doi:10.1128/mbio.03890-25)
Supplement: Supplemental material — Supplemental figures and tables. [file mbio.03890-25-s0001.pdf]

Supplemental Material

A bacterial family of fatty acid acyltransferases related to the *Shigella* effector IcsB

Waad Bajunaid[a,b], Kyle Tomaro[a,b,c,d], Anwer Hasil Kottarampatel[a,b], Geneviève F. Desrochers[a], Mathieu Lavallée-Adam[d], John P. Pezacki[a,c] and François-Xavier Campbell-Valois[a,b,c,#]

[a] Department of Chemistry and Biomolecular Sciences and Centre for Chemical and Synthetic Biology, University of Ottawa, Ottawa, ON K1N 6N5, Canada

[b] Host-Microbes Interaction Laboratory, University of Ottawa, Ottawa, ON K1N 6N5, Canada

[c] Department of Biochemistry, Microbiology and Immunology and Centre for Infection, Immunity and Inflammation, University of Ottawa, Ottawa, ON K1N 6N5, Canada

[d] Department of Biochemistry, Microbiology and Immunology and Ottawa Institute of Systems Biology, University of Ottawa, Ottawa, ON, Canada

Running Title: Identification of a family of IcsB-like acyltransferases

#Address correspondence to François-Xavier Campbell-Valois, fcampbel@uottawa.ca

Table S1. Information about the IcsB homologs represented in Figure 1A in order of appearance from the top to the bottom of the tree.

Homolog[1]	Sequence ID	Species	Class[2]	Isolation
	WP_059887048.1	*Burkholderia ubonensis*	Beta	Soil
H1 (BopA)	WP_004528810.1	*Burkholderia pseudomallei*	Beta	Soil
	WP_175743736.1	*Burkholderia ambifaria*	Beta	Plant
	WP_060235254.1	*Burkholderia ubonensis*	Beta	Soil
H3	WP_048412814.1	*Chromobacterium* sp. LK11	Beta	Plant
	WP_271787565.1	*Aeromonas salmonicida*	Gamma	Fish
	WP_080428163.1	*Burkholderia ubonensis*	Beta	Soil
Burkholderia clade: similar proteins found in *B. oklahomensis* ,*B. thailadensis* and unclassified species, *C. alkanivorans* and *C. rhizoryzae.*				
H9	WP_088447978.1	*Achromobacter denitrificans*	Beta	Soil and water
H6	WP_043548505.1	*Achromobacter* sp. Rta	Beta	Termite gut
H4	EHK65445.1	*Achromobacter arsenitoxydans*	Beta	Arsenic-contaminated soil
Achromobacter clade: similar protein in *A. spanius and A. xylosoxidans* str. JCM 9787				
IcsB	WP_031192845.1	*Shigella flexneri*	Gamma	Clinical sample
H2	WP_038355842.1	*Escherichia marmotae*	Gamma	Faeces of *Marmota himalayana*
IcsB clade: *Shigella* and related *Escherichia* spp. with pINV or related invasion plasmid				
	WP_153765867.1	*Endozoicomonas* sp. OPT23	Gamma	Colonizes *Ophlitaspongia papilla*
	WP_153765866.1	*Endozoicomonas* sp. OPT23	Gamma	Colonizes *Ophlitaspongia papilla*
H7	WP_034879418.1	*Endozoicomonas montiporae*	Gamma	Sea water
Endoizocomonas clade: similar proteins in other *Endoizocomonas* species.				
	WP_024901981.1	*Robbsia andropogonis*	Beta	Leaves
H8	WP_024901980.1	*Robbsia andropogonis*	Beta	Plant
	WP_024901979.1	*Robbsia andropogonis*	Beta	Leaves
Robbsia clade: mainly *R. andropogonis* with some strains possessing all three proteins represented in the tree				
	WP_139446239.1	*Desulfobotulus mexicanus*]	Delta[3]	Lake sediment
H10	WP_008870026.1	*Desulfonatronospira thiodismutans*	Delta[3]	Lake sediment
H11	WP_054022033.1	*Ideonella sakaiensis*[3]	Beta	Sediment in a plastic recycling facility
Environmental clade				
H5	WP_025424429.1	*Sodalis praecaptivus*	Gamma	Human wound
Sodalis clade: limited to *S.praecaptivus* and poorly characterized *Candidatus Sodalis pierantonianus*				

[1] Homolog number of selected proteins as described in Figure 1.
[2] Classes in the Pseudomonadota phylum; beta-, gamma- and delta-proteobacteria.
[3] *D. mexicanus* and *D. thiodismutans* have recently been reclassified in the newly created phylum Thermodesulfobacteriota.
[4] Also known as *Piscinibacter sakaiensis*

Table S2. Prediction of the occurrence of non-flagellar T3SS in the genome of bacteria encoding the selected IcsB homologs.

Protein	NCBI Reference Sequence	Ensembl Genome Assembly	# of Predicted T3SS
H1	NC_006351.1*	BP_3921g	3
H2	NZ_CP025980.1*	NA	1
H3	NZ_LDUR01000009.1*	ASM104370v1	1
H4	NZ_AGUF01000055.1	ASM23678v2*	1
H5	NZ_CP006569.1*	ASM51742v1	3
H6	NZ_JPYO01000036.1*	ACRTA	1
H7	NZ_CP013251.1*	ASM72256v1	1
H8	NZ_CP139170.1*	ASM97034v1	3
H9	NZ_CP020917.1*	NA	1
H10	NZ_ACJN02000001.1	ASM17443v1*	0
H11	NZ_BBYR01000069.1	ASM129352v1*	0

*This indicates the sequence used in the prediction method. First, NCBI was used. Occasionally, TXScan failed to return a result, likely due to annotation issues. This was resolved in all cases by using data from the ENSEMBL Bacteria database.

Table S3. T3SS substrate predictions for the selected homologs of IcsB with DeepSecE.

Protein	DeepSeqE	Score
IcsB	Yes	0.8940
H1	Yes	0.9895
H2	Yes	0.9654
H3	Yes	0.9993
H4*	Yes	0.9984
H5	Yes	0.9752
H6	Yes	0.9906
H7	No	NA
H8	Yes	0.9947
H9	Yes	0.9982
H10	No	NA
H11	No	NA
RecA	No	NA

IcsB and RecA are used as positive and negative control, respectively.
*Obtained with an alternatively annotated H4 (WP_043518452.1) that extended the N-terminus by 16 residues compared to EHK65445.1. The longer version is of similar length to H6 and H9.

Table S4. Structure prediction of IcsB and its homologs with palmitic acid by AlphaFold

Protein	PTM#	iPTM*	Docking of palmitate in the catalytic site (Yes/No)¶
IcsB	0.78	0.82	Yes
H1	0.71	0.8	No
H2	0.78	0.82	Yes
H3	0.73	0.81	Yes
H4	0.65	0.86	No
H5	0.59	0.5	No
H6	0.65	0.71	No
H7	0.68	0.7	Yes
H8	0.66	0.79	Yes
H9	0.72	0.84	Yes
H10	0.81	0.81	Yes
H11	0.8	0.82	No

AlphaFold3 predicted template modeling (TM)-score for the full structure
*AlphaFold3 predicted interface template modeling (TM)-score for the full structure
¶ To be considered properly docked the carboxylic group of the palmitic acid had to be inserted in the catalytic site in the vicinity of the cysteine nucleophile (C306)

Table S5. Mass spectrometry raw data, fold change enrichment and statistical analyses of proteins retrieved from IcsB and toxic homolog samples.

See corresponding Excel file attached with this manuscript.

Table S6. Acylated proteins based on the mass spectrometry data used in the upset analyses.

See corresponding Excel file attached with this manuscript.

Table S7. Plasmids used in this study.

Plasmids*	Origin	Addgene number
pRS313	Gift from Cammie Lesser	NA
pRS313 GAL1p2	This work	242948
pRS313 GAL1p2 IcsB	This work	242949
pRS313 GAL1p2 H1	This work	NA
pRS313 GAL1p2 H2	This work	NA
pRS313 GAL1p2 H3	This work	NA
pRS313 GAL1p2 H4	This work	NA
pRS313 GAL1p2 H5	This work	NA
pRS313 GAL1p2 H6	This work	NA
pRS313 GAL1p2 H7	This work	NA
pRS313 GAL1p2 H8	This work	242950
pRS313 GAL1p2 H9	This work	NA
pRS313 GAL1p2 H10	This work	NA
pRS313 GAL1p2 H11	This work	NA
pRS316	Gift from Cammie Lesser	NA
pRS316 GAL1p1	This work	242951
pRS316 GAL1p1 IpgA	This work	242952

*For simplicity, plasmids harboring acyltransferase catalytic mutants akin to IcsB H145A, D195 and C306A are not indicated in this table. Details for their generation are indicated in the Materials and Methods.

Table S8. Primers used in this study.

Primers	Sequence 5'- 3'
Gal1p_Sp6_S	ACACTATAGTAAGTGACGCTGACTAAC
Gal1p_Sp6_R	CACCTAAATAGAGGTATATTAACAATTTTT
Gal1p_BGH_S	TGCCTTCTATCATGTAATTAGTTATGTCAC
Gal1p_BGH_R	CAGTCGAGGTCGGCCCGTGGCTCAGCTGC
Gal1p2_3xFlagBGH_F	AAGGAGGCCGAATTCC
Gal1p2_3xFlagBGH_R	GTAAGCGTGACATAACTAATTACA
IpgA_S	AGAGGAATTCGCTAGGAGAAATTAACCATGTGTCGCAAACTATATGATAAACTTTATG
IpgA_R	AGAGAGGGATCCGTTCACTTCTGAAGTGATGTTTGC
IcsB_C306A_S	GCCGCTGGTATGGCACTTAATGTTC
IcsB_305_R	GTTTTCAGATTTACTGATTAATTTATA
IcsB_H145A	GCCACTAGTATCAGTATAAAAAACAATTTACTACA
IcsB_144_R	CCCTAAATGGGTTATGCCTC
IcsB_D195A_S	GCCAAACTAAATATGATTTCAGAGCAAACAGC
IcsB_D194_R	TTGCTTATAGGTATCGACACTGTAAC
H1_H144_S	GCCGCTTCATTAACTATTAAAAATCAA
H1_143_R	ACCTGGATGATCCAAAC
H2_H145A_S	GCCACTTCCGTTAGCGTGAAAAACA
H2_144_R	GCCCCAATGGTCCGCCCCC
H3_H146A_S	GCCGCTTCCGTCACAATTAAGAATAA
H3_145_R	GCCCGGATGGTCCATC
H4_H122_S	GCCTCAGCCGTCGCGATTTC
H4_121_R	TCCTGAACGCTGAATGTT
H5_H176A_S	GCCGCCGCACTTGCACTGAGTACC
H5_175_R	TCCACAGTGATTGGGATCGTG
H8_H157A_S	GCCGTAGCATTGCTGATGAGGC
H8_156_R	TCCGACACGTCTCATATT
H10_H23A_S	GCCGCTGCATTGAAGATCAGGACGAA
H10_22_R	CCCCGGGGACTTCATTCCA
H1_D193A_S	GCCAAAAGGTCAGAAATTTCTTCTG
H1_192_R	AATTCTATAACCATCCAAAG
H2_D193A_S	GCCAAGCTTAACATGTTGTCAG
H2_192_R	CTTACGATATGAGTTAGTGCT
H3_198A_S	GCCAAGCAGGAGGAGGTATCAAG
H3_187_R	CTCCCTGTAGGAACTAAGGG
H4_D185A_S	GCCAGAGGAGACGAGATTTCAGAG
H4_184_R	TGAATCGTAATCCGGTGCTG
H5_D221A_S	GCCACTTATGACAGCTTGAGTGAGACC
H5_220_R	CTGCGGATAATGAGCGA
H8_D228A_S	GCCATGTACTCAGAAATGAGCCAAAGG
H8_227_R	AGATGTGTAACTCCCCGCG
H10_D69A_S	GCCAAGTTCTTAGAGATGAGTGCCTC

H10_68_R	TTTGTAATAGCCTGGTTGGG
H1_C312A_F	GCCGCATCTATGGCTTTGAGAGTTTT
H1_311_R	ATTTTCTTTTTTAGAAATCATTTGATAC
H2_C307A_F	GCCGCAGGCATGGCGTTAGAC
H2_306_R	GTTTTGGGACTTACTGATTAGTT
H3_C317A_F	GCCGCGTCCATGGCGCTACGTATG
H3_316_R	ATTTTCCTCCGTTGACGCAAGCGTATA
H4_C332A_F	GCCGCCGCAATTGCAGCTCGTTCA
H4_331_R	ATTCTGTGTGGTGGAGAACATGCTG
H5_C442A_F	GCCTCCGCGATGGCCCTACGTTCC
H5_441_R	GTTGTGACGGGTGCTAACCAGACG
H8_C341A_F	GCCAGTGGCATGGCTGCCAGGTTGTTA
H8_340_R	ATTCCTGTCTGTTGAGACCAGCTTG
H10_C184A_F	GCCGCCGCAGTCGTGGCACAA
H10_183_R	GTTATACTTTGTTGAAACAAAACTGTAGCC

```
IcsB/1-494          MSLKI--SNFI--D-A-SN--------TKGPIRVEDTE---------------HG----P
H1/1-512            MIN-V--DAFV--A-S-AR--------SGARVVVGGDA---------------RG----P
H2/1-492            MNMKV--SDFN--I-A-SN--------MTGRINIEKSE---------------EG----Y
H3/1-516            MTT-LNLNTFV--Q-A-AR--------LGGRVVINEKT---------------PE----P
H4/1-677            MRL-D------------------------------DSG---------------SA----A
H5/1-622            MIT-L--QAFD--G-AYSA--------QNGPQALQIAP---------------ASANGIP
H6/1-687            MVQ-L--SSFL--S-A-SV--------QGGRVQLDDTA---------------TN----A
H7/1-503            MAG-F--DIYV--F-K-SRADAANLAAQVSRVQKEVKG---------------EN----A
H8/1-520            MTH-I--PLAPGGAVA-DL--------QAGWEAVTDVRPFAEAERQSNLIEHGFS----P
H9/1-687            MVQ-L--SSFL--S-A-SV--------QGGRVQLDDAA---------------QT----A
H10/1-375           MRI---------------------------------------------------------
H11/1-368           MQL---------------------------------------------------------

IcsB/1-494          ILIAQKF-NLKDLFFRTLSTINAKINSQILNEQLKNYRLEN---QKSLLLFLNTLASEKS
H1/1-512            VVSAARL-GMKERLFAFLAHVPLLKHCDAVRRYAEQVRMEN---RRSLEVFVLALSKRYG
H2/1-492            KLISSPF-SLKDLCFKTLSKINSKLHSKELDSMLKNYHIDN---QKAVLQFLNALAKEKS
H3/1-516            TVTIASQ-GFKGRLYACLSQLPLLKNLEAVKSYSQRVQAEN---QTALGVFINTLSHRYG
H4/1-677            Q----GR-GVVGR----LKDWIV---SLPGGQGAESVKQDN---MKATAVFAQALAGEYG
H5/1-622            AFVARRR-EIIDILLEYLARVRQFRDIDFVARYIRRTDQEN---QRLKQHLVSALQSVNT
H6/1-687            Q----GR-GMFGR----LKDRI--------VQGSEQVKESN---HRANSVFAQALAREYG
H7/1-503            G----FLSVSWAK----LKSGI--------TRQV-KIPASN---SKAE------------
H8/1-520            L----WR-GVVRL----LSSLPIIGNTNPVKQAAADITFSDTARAERFEAFVGALRSQYS
H9/1-687            Q----GR-GVFGR----LKDRI--------VMGSDQIKESN---LKANMVFAQALAGEYG
H10/1-375           ------------------------------------------------------------
H11/1-368           ------------------------------------------------------------

IcsB/1-494          AESAFAAYEAA-K--------NSI---QHSFTGRDIKLMLNTAEAFH-GIGTAKNL«ERHL
H1/1-512            PEGAKAAFDYG-A--------RRD---GAPLDQRRVRNMVSIAEHFH-GTGDAKPL«ARQM
H2/1-492            FESTFFAYEAA-K--------SRL---QHSFTGKDIKTMLNAADRFH-GIGTAKNL«ERHL
H3/1-516            QESAQAALDSM-G--------RLQ---GAPLKQRVVEQLISVAERFH-GQGDAKPL«ARQV
H4/1-677            FKAAAAAVDRVIG--------RNF---DAALDKAKIDKMVSVAQGLS-GLGAARSL«ARNV
H5/1-622            PP-GQAKIDYALAPWFSPEYHPTP---GRPLKCKDIDEIQDRLARFD-GVGDARES«SRRL
H6/1-687            YKAAAAAVDRVIG--------RDY---TATLNKTKIDKMISVAQGLS-GVGKARDQ«ARNV
H7/1-503            ----MSAMKPVVS--------NLQELKDKELCTR-KASPFDVPEGFSWSHGNAKRM«SRHI
H8/1-520            AEITDEVVAL-TA--------LSP---ANYLSLSKMREARDVALMRE-CLTHNQQF«TNRI
H9/1-687            HKAAAAAVDRVIG--------RDY---TATLDKAKIDKMVSVAHGLS-GMGKARDQ«ARNV
H10/1-375           -------------------------------------------------------Y«ESEA
H11/1-368           -------------------------------------------------------Y«EHEV
                                                                             .

IcsB/1-494          VFRCWGNR----GITHLGHTSISIKNNLLQE--------PTHTYLSWYPGGNVTK-----
H1/1-512            VFRSWECR----GLDHPGHASLTIKNQADAD-----AGRHVYEHVSWWPNQRLG------
H2/1-492            VLRCWNYE----GADHWGHTSVSVKNNMKPE--------PSHMYLSWYPLNNSTK-----
H3/1-516            VFRSWECK----GMDHPGHASVTIKNKIDVN-----ANKHVQEHISWWPFSDAQG-----
H4/1-677            CLNSWARESSGLNIQRSGHSAVAISNGLSAN-----TWSHEKQYVSWWPSTNDSVQ----
H5/1-622            TVNIWPYH----DPNHCGHAALALSTP------------ERQAYFSYWPDGELPD-----
H6/1-687            CLNSWPRVSSGFNIQRTGHSAVAISNTMSPN-----SWSHAKEYVSWWPAKSDVE-----
H7/1-503            VVRHWTTP--G-KIDSSMHTALSMKDKIA----------DIDEYATWTPRKIRLINWSRS
H8/1-520            EVKVWNWE----NMRRVGHVALLMRHELTDGGDTKLTGPDAETYASWWPGGDFDAS----
H9/1-687            CLNSWPMVTSGLSVVRSGHSAVAIANTLSAN-----AWSHGKAYVSWWPAKSDVQ-----
H10/1-375           EVLIWPYH----GMKSPGHAALKIRTNKDGE--------VYQTYVSWWPAGGEGK-----
H11/1-368           LVYVWRFS----GF-RTGHASIKLKAPGLLNP---AANGKQHQYVSWWPRGGPND-----
                    .   *             *: : :                   : :: *
```

```
IcsB/1-494     -DT----------EINY--LFEK-RSGYSVDTYKQDKLNMISEQTAERLDAGQEVRNLLN
H1/1-512       --------------SKE--HFDR-IEPKTLDGYRIDKRSEISSATEQRLREGDAARRKIL
H2/1-492       -IT------------ST--YFSK-SLSISTNSYRKDKLNMLSDRTVQRLNAGEEYKKSNE
H3/1-516       -G-----------QIGR--LFGQ-RQGGSLSSYREDKQEEVSSRTAQKLSDGEDARQQLG
H4/1-677       -VDRNRLLEKL-PVVGD--YFHA-RPAMSAPDYDSDRGDEISEKTNINLQRGEAAREVLR
H5/1-622       -G-----------NKQR--YLGP-RPAQLVAHYPQDTYDSLSETTRKKLTEAHVMRGLIR
H6/1-687       -VSRNRALEML-PGVGG--HFEA-RPGMSAPSYDSDRGDEISEKTNLNLQRGQAARDVLK
H7/1-503       KNPFKRFLAPIKMKLDD--LLTQ-DFPIAPPSYRDDKALYLGDRTKFRLQAGVDARQSIA
H8/1-520       -PDEGQVSGLE-AKISDRLEFDIQTQRGFAGSYTSDMYSEMSQRAREGLESGRFAPLPGQ
H9/1-687       -VAHNRLLASL-PGVGR--HFAA-RPGMSAAGYDSDRGDEISKDTNRNLQRGQVARDILR
H10/1-375      ---------------NT--PFSY-RPAEAQPGYYKDKFLEMSASTRNRLNDGEINRDPRR
H11/1-368      ---------------PG--GLRW-RDAAPSTSYRSDRREELSPKTRQALASGRFKAM-GR
                                   :           *  *    :.  :   *  .

IcsB/1-494     SK----------------------------------------------------------
H1/1-512       ADGF--------------------------------------------------K---YA
H2/1-492       HE--------------------------------------------------------IV
H3/1-516       TAGY--------------------------------------------------K---AA
H4/1-677       SAARLAS--EGRADPLADALKK------------H--------ADDLKGLEFGDN---PT
H5/1-622       EGEEESFLWEHVEDRLEDIAKYINLSKKDYNDQDIYILKKLIKSMPLKPVNYVMTATQRV
H6/1-687       TAARLER--AGDKDPLGATRQE------------H--------AEELGELGFDED---VT
H7/1-503       EKEAVNP--LIDRH-IEVTVPE------------T--------VLPQAD-G-GQD---EI
H8/1-520       VP----------------------------------------------------------
H9/1-687       AADGFTH--DRKSDPLGAALQA------------H--------AEALDGLGFGEG---VT
H10/1-375      ------------------------------------------------------------
H11/1-368      Q-----------------------------------------------------------

IcsB/1-494     ------------------------------------------------------------
H1/1-512       N-----------------------Q-----------------------------------
H2/1-492       K-----------------------------------------------------------
H3/1-516       S-----------------------Q-----------------------------------
H4/1-677       R-----------------------K-----------------------------------
H5/1-622       KQDYGNALLIKTDVIFCQAIVTTMAEQSNAGRLTEKEISKIVAGRLRQMPTFCDQLQNNI
H6/1-687       L-----------------------Q-----------------------------------
H7/1-503       T-----------------------D-----------------------------------
H8/1-520       ------------------------------------------------------------
H9/1-687       R-----------------------E-----------------------------------
H10/1-375      ------------------------------------------------------------
H11/1-368      ------------------------------------------------------------

IcsB/1-494     QDQNNNKKIFFPRANQKK--D-PYGYWGVSAD-K-V---------------------YIP
H1/1-512       -DE-RHDARFFPRAGQKL--D-KDAEWGLSAR-K-V---------------------YFP
H2/1-492       TDI-NRKDALYPRANQKK--DIDQASWGVSAN-K-I---------------------YIP
H3/1-516       -DL-MAKAQYYPRAEQKR--T-RDGGWGVSAR-K-V---------------------YLP
H4/1-677       -DL-ESAAKFFARDSQEL--V-SDRQWGAAAE-K-V---------------------FFP
H5/1-622       ARA-QCLAGFQPRSRQVW--DEENDAFVATSD-Q-I---------------------YLP
H6/1-687       -DL-QAAARFFPRDSQEL--V-SGRTWGAGAE-K-V---------------------FFP
H7/1-503       -NT-VKTANYKPLPFQKTSSK-DNREWQRRAE-K-H---------------------YLP
H8/1-520       --------INIY-QGGEDE--A-PNVSWGKEPDAS-I---------------------AMP
H9/1-687       -EL-QAAAGFFPRDSQER--V-SDRTWGAGAE-K-I---------------------FFP
H10/1-375      ----------FQKVSQNP--NT-ALEFVTSAN-KKFNLPGLFSRVGRTRKSGAGIDGFDN
H11/1-368      ----KASLPYEPKLGEDP-LT-DANAWGLSADAK-I---------------------RLP
                              :          :   .
```

```
IcsB/1-494     LSGDNKTK-D---------G-KISHNLFGLDETNMSKFICKKKADAFRQLANYKLISKSE
H1/1-512       AIGFNHDRRD--------TDRPRAFVLFGLNEAAMLRDARTVKEGAKSGELMYQMISKKE
H2/1-492       LQGENQSR-D---------G-GTEYNLFGLDESKLSSFICKKRGGAFRRTEHYKLISKSQ
H3/1-516       MMGRNKEVAA--------SGKTAKFVLFGLNEKAILHEARHVKQEAAEGRIGYTLASTEE
H4/1-677       LAGRNGEHTA--------QGLIPQTTLFGLVEHDMLADARQLKDDAAHGRIGYSMFSTTQ
H5/1-622       IAGE-------------------RAELFGLDEQALEEFWLSVKSEIATGKARFRLVSTRH
H6/1-687       LAGRNGEHGS--------GGLQPRTTLFGLAEHDMLADANQLKADAEQGLIGYSKLSTTQ
H7/1-503       CVGFDKDQWT---------G-RETFTMFGLDLEKMRNKWIAVKN-PEHPNHYYKQFSTEQ
H8/1-520       MAGYNERPHPGGTGTDANGVVVPTLTTFGLSVDAIKTYWET-E-AIGNDAAQFKLVSTDR
H9/1-687       LAGRNGEHGN--------DGVSPRTSLFGLAEHDMLADANQLKSDAAQGRIGYSMFSTTQ
H10/1-375      VAGRSSLA-------------DGEYTYWGLNLPGMVKWWMD----FSDTPKGYSFVSTKY
H11/1-368      GLGNAG-------------------LVFGLDIGAMVRWWNLFTH---SGSNEFN--ILKL
                                          :**    :                 :

IcsB/1-494     NCAGMALNVLKAGNS»EIYFP-LPDVKLVATPNDVYAYANKVRQRIESLNQSYNEIMKYIE
H1/1-512       NCASMALRVLRAGGA»EHFVP-YTAAWISEDPNHAHAYALAVQARIDALNQRRADVERRCE
H2/1-492       NCAGMALDVLKAGDA»ETYVP-FPKIKLVANPNDAYKYAYKVMARIEELNKIHKGIIKNVD
H3/1-516       NCASMALRMLRAGGA»ENFVP-FSASWVTEDPNKAHAYAERLQARMDSLNRQVADIGERCS
H4/1-677       NCAAIAARSLQAGGS»NIYVP-FEASWITEDPNKTYDYARQLQGAIDGLNGKADGIKKFCD
H5/1-622       NCSAMALRSLQAAGA»GRYVP-LPARWLLYTPRDVEHYTLRLQKALDRLNRQSSAIQAFYE
H6/1-687       NCAAVAARSLQAGGS»NIYVP-FEAAWITEDPNKTYAYALQLQAAIDQLNDQAGSVKAHCA
H7/1-503       NCSGMCLSLLKEGGA»GLFYN-FSPSLV-TTQSDVEKYSAKLVDKLDRLNKHVDDLDEKIR
H8/1-520       NCSGMAARLLKAGGA»HAFLP-VPEAVLFLDPNTMRRYTTDLMTEVTALNAKADTVDASFG
H9/1-687       NCAAVAARSLQAGGS»NIYVP-FEASWISEDPNKTYDYARELQGAIDLLNEQEGSVKAHCA
H10/1-375      NCAAVVAQCLLEGCA»DAYID-IKQPKMYYTPNDISALADKLVNRLDDLNIESRRLINNSR
H11/1-368      NCSRVAAMCLLAGGA»ARYCKPPTRAFNLWTPNSIEAWALKLDSALKARNEAAKEIELERL
               **: :    *  . :   :                   :  :   :   *     :
IcsB/1-494     S«DFDLSR-LT---------QLRRSYLKSFNKINLIHTP----------------------
H1/1-512       R«LRDSAS-VRQA---------WRAFSEAGGA--------------SA----S--------
H2/1-492       E«KIDIMT-MI---------KLRRSYLNSFNKVTSSHKI----------------------
H3/1-516       G«MLQQPG-VKTE---------WDAFLNQGRM--------------------G--------
H4/1-677       T«AWQSAP-ADADA----IRQLRDALHETANDQAIFAQAKQLALGIAADQRIDSLRAEVAD
H5/1-622       Q«ETASR-------------------------PNDLA------------------------
H6/1-687       A«AWSKAP-AEADA----ISILRATLLVRADDLDAYDQTRQQAQGVAADQRILGLSADVAH
H7/1-503       L«Y--KVP-NEPELPLSSIPE-KLVFLLSTYSM----------------------------
H8/1-520       R«LFTEDEGADESG----VTPSYSVRLARA-------------------------------
H9/1-687       S«AWSKAP-AEADT----IAQLRVALHARAHDLDAYDQTRQQAQGTAADQRIRGLGADVGH
H10/1-375      Q«SLDRPG-IWTS---------EEFYK-ASY-AGRFA------------------------
H11/1-368      P«SNDQPP-SYL---------LDVGTWQKATKPGSFSVRRGQVL--KIDKLLHEYGVVAGR

IcsB/1-494     ---------KTFKP--LSISLYKHPT--------------------EN------------
H1/1-512       ------PLAEDAGRGRAS-----AHM--------------------RQ------------
H2/1-492       ---------NKFNT--LLVIRNKDNI--------------------EN------------
H3/1-516       ------ELKREISQ--LK-----QQINKER--------RPEAQAVQRQ------------
H4/1-677       RSGKMDQARSLLQP--LD-----RQIRATEKDVGDRQKAVD------NKQKAVDGMSRSA
H5/1-622       ------IR---QSD----------------------------------------------
H6/1-687       AKGRVQEMEQELAG--LE-----TKIVATQKEVNGRSEKVR------LAADSLAARAARL
H7/1-503       -----------------D-----E------------------------------------
H8/1-520       --------QEAIAS--LP-----R------------------------------------
H9/1-687       ARDNLQKAERELKG--LE-----NTIPAMEKEVAARSDRIN------RTAASLEARAENL
H10/1-375      ------RRKGQVAS----------------------------------------------
H11/1-368      TD--AEGQREALNV--LE-----QILGQVHSHIVQKGD----------------------
```

```
IcsB/1-494     ------------VSSEDFDAVIN-----------------AC-----------------H
H1/1-512       ------------ARLDEHAREVE-----------------RIG-----------------
H2/1-492       ------------ASLDDYNKIIN-----------------SC-----------------F
H3/1-516       ------------ALKERNAQQVF-----------------RIG-----------------
H4/1-677       KSSSPHAAARLADSKAELAERQDELKKGQAELNDLRGQRSRLDTRILAMKDERRALKDLI
H5/1-622       -------------------------------------------------------LTGLR
H6/1-687       QPDDAKAAAKLNADRKSHQKDLAALELRRGELATQLALKPRLEQRILALDEDHEALSDLL
H7/1-503       -----------------------------------------------SWKAKIQEVASII
H8/1-520       -----------------------------------------------ATQASMQALNNAL
H9/1-687       NPENTKEVVKLKADRDSHDKNVNALNRRREELSAQVGRKARLEQRIPALRDEHEALSGLL
H10/1-375      -------------------------------------------------------IDDLL
H11/1-368      --------------GSRNREV-----------------VLQLGQHVLKLYRERSVRLALL

IcsB/1-494     SY------------------------------------------------LVKSAPSN--
H1/1-512       --------------------AYFAELSAGRSGK-----------------HRDRADAA--
H2/1-492       ES------------------------------------------------LKKLAPHN--
H3/1-516       --------------------AHFEQLAVGRA--------------------D---ALA--
H4/1-677       EER-QANPLEAARRKLGGEDDYYAKLRGGLAEGVTGLAHAFGARASGLDTQQQRLLAP--
H5/1-622       TQFNLL--------------------------------------FSHLQTPQQKRLRRLA
H6/1-687       EDR-KAFPVEAAQLRPAGEEGYYAAQRRGLQSCVEGLADQFDAHIQGLDARQQRQLAP--
H7/1-503       HE----------------------------------------------------------
H8/1-520       NE----------------------------------------------------------
H9/1-687       NDR-TAFPVEAAQLKLPGEEGYYAAQRRGLQSCVDGLAQQFDARLQGLDARQQRQLAP--
H10/1-375      KEYHKAGPWD---------ENNFPQKFKALGRMM----------RNVLDHRQKKAGSDRG
H11/1-368      KQS-QAKNLVGAS-KYQLLDEYNALMAEGLKLGTESWGQ---------------------

IcsB/1-494     -------------MTRVLNELK--TEATDKKEEIIEKSIKIIDYY---------------
H1/1-512       -------------LADAMKRCA--PSARDDVAALTRKASVLVETL---------------
H2/1-492       -------------SHSRISEFK--PTMTDTKEQLIEKSIKIIDFY---------------
H3/1-516       -------------LAKAMREGA--PSIDDDAAVLTRKTAGLVEAL---------------
H4/1-677       -------------LGQAVQDLERGVRNGSDMKTLLSHAKQLVQTL---------------
H5/1-622       RAVNT---------------LPDAFLSQQS-DRIIPAAKALVEALSPLLTDAISQQQDYR
H6/1-687       -------------LGRAVSELRDAVNAGGGMQALLSKAKPLVETL---------------
H7/1-503       --------------------IENAPSTLKGLTPIAIRLTTSLDRL---------------
H8/1-520       --------------------CK---TEAGDYEGMMRQMKAMVGGL---------------
H9/1-687       -------------LSGAVADLRAAVDAGAGMQALMSKAKPLVETL---------------
H10/1-375      GAVETLGLQILAELSSEELLIAQNNNAGGS------------------------------
H11/1-368      ------------------------------------------------------------

IcsB/1-494     -------------NSLK------SPDLG---------------T--KLYIHDL---LQIN
H1/1-512       -------------GRHLD-APP-PSDSS---------------ALRRLAAHAM---IGRI
H2/1-492       -------------QSLK------NKNQE---------------L--LFYTHDL---LLVN
H3/1-516       -------------HHYQE-SHP-QGAEQ---------------L--SLAAHAM---IKRS
H4/1-677       -------------HDAIN-GTP-APDRV---------------A--ALAAGAL---VQAC
H5/1-622       TTLSLAAAMRTWLQQRQQRGVP-S--------------------------SAL---YSAA
H6/1-687       -------------HALTS-GAP-AADQI---------------A--ILAAGSL---VEAC
H7/1-503       -------------FKITS-DNPYLTDRL---------------E---PALHAFKILKNRM
H8/1-520       -------------HNHVN-GPF-GSDINKALRSAYELYSAIRGA--AHEAGYL---REAS
H9/1-687       -------------HALKD-AAP-AADLV---------------A--MLAAGTL---VEAC
H10/1-375      --------L-----------------------------------------AAL---LSLC
H11/1-368      ------------------------------------------------------------
```

```
IcsB/1-494      KLL--LNN--SH--SNI--------------
H1/1-512        EAF--MAA------AI-A------------A
H2/1-492        KTL--LDK--S-----R--------------
H3/1-516        EEL--MGL------AI--------------Q
H4/1-677        EFL--VVN--DQ--DR-HS------------
H5/1-622        SAT--SQR--DH--HRAHYARKYAVPDSPER
H6/1-687        EIL--ITL--DQ--DR-RA------------
H7/1-503        EDA--YREQVEMFEDP-YF-----------E
H8/1-520        DSD--IPS--DF--FD--N------------
H9/1-687        EVL--VTL--DQ--DQ-HA------------
H10/1-375       EEGNNRTL--DQ--SF-KKAKKF--------
H11/1-368       -------------------------------
```

Figure S1. Multiple full-length sequence alignment of IcsB and its selected homologs. Full-length sequences were aligned with T-Coffee and analyzed with Clustal Omega. The residues forming the catalytic triad are white with black shading. The star (*) and dark gray shading indicate full conservation. The colon (:) and point (.) with light gray shading indicate decreasing levels of conservation. The catalytic domain used to construct the phylogenetic tree is the region within « » (residues 127-319 of IcsB). This catalytic domain and its C-terminal extension within » « constitutes together the extended catalytic domain (residues 127-364 of IcsB) that was used for the RMSD calculation. It is noteworthy that there is a poorly conserved insertion a few residues after the catalytic residues D195 that is indicated with regular, non-bold lettering. The poorly conserved N-terminal and C-terminal domains are indicated with gray lettering. Related to Figure 1 and Table S1.

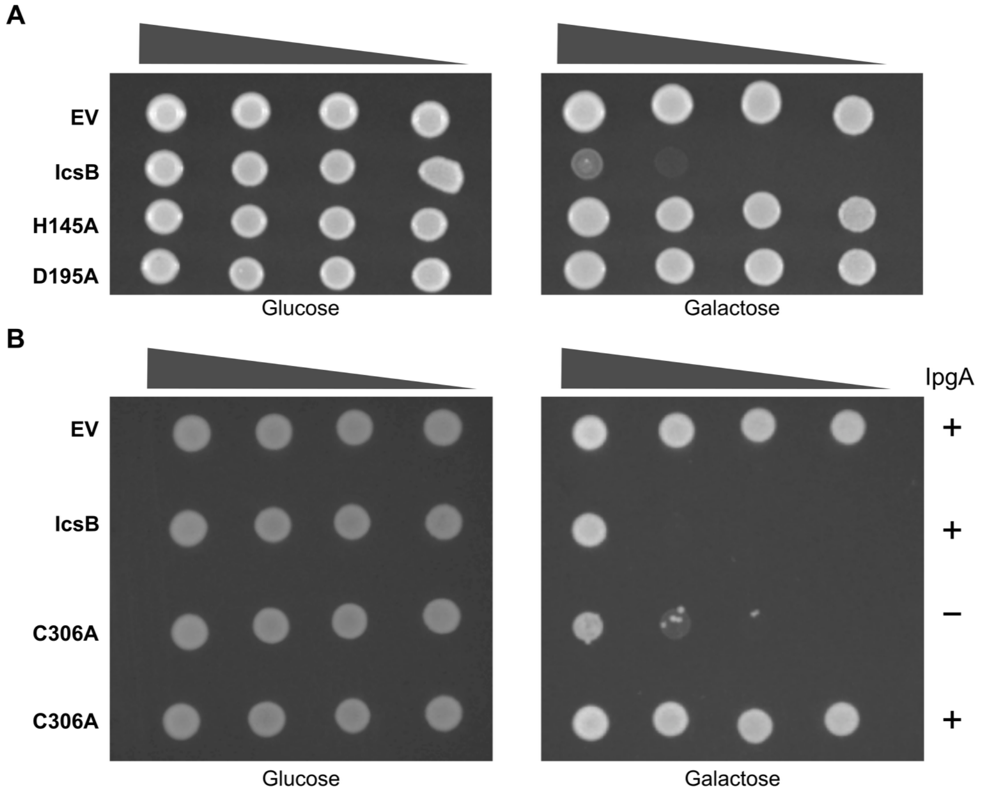

Figure S2. Validation of the toxicity assay with IcsB. (A) Expression of IcsB under a galactose inducible promoter inhibits yeast growth on solid medium. H145A and D195A that disrupt the catalytic site rescue cell growth. (B) The rescue of the growth inhibition phenotype by C306A, which disrupts the third catalytic residues, is null unless it is co-expressed with the IcsB chaperone IpgA.

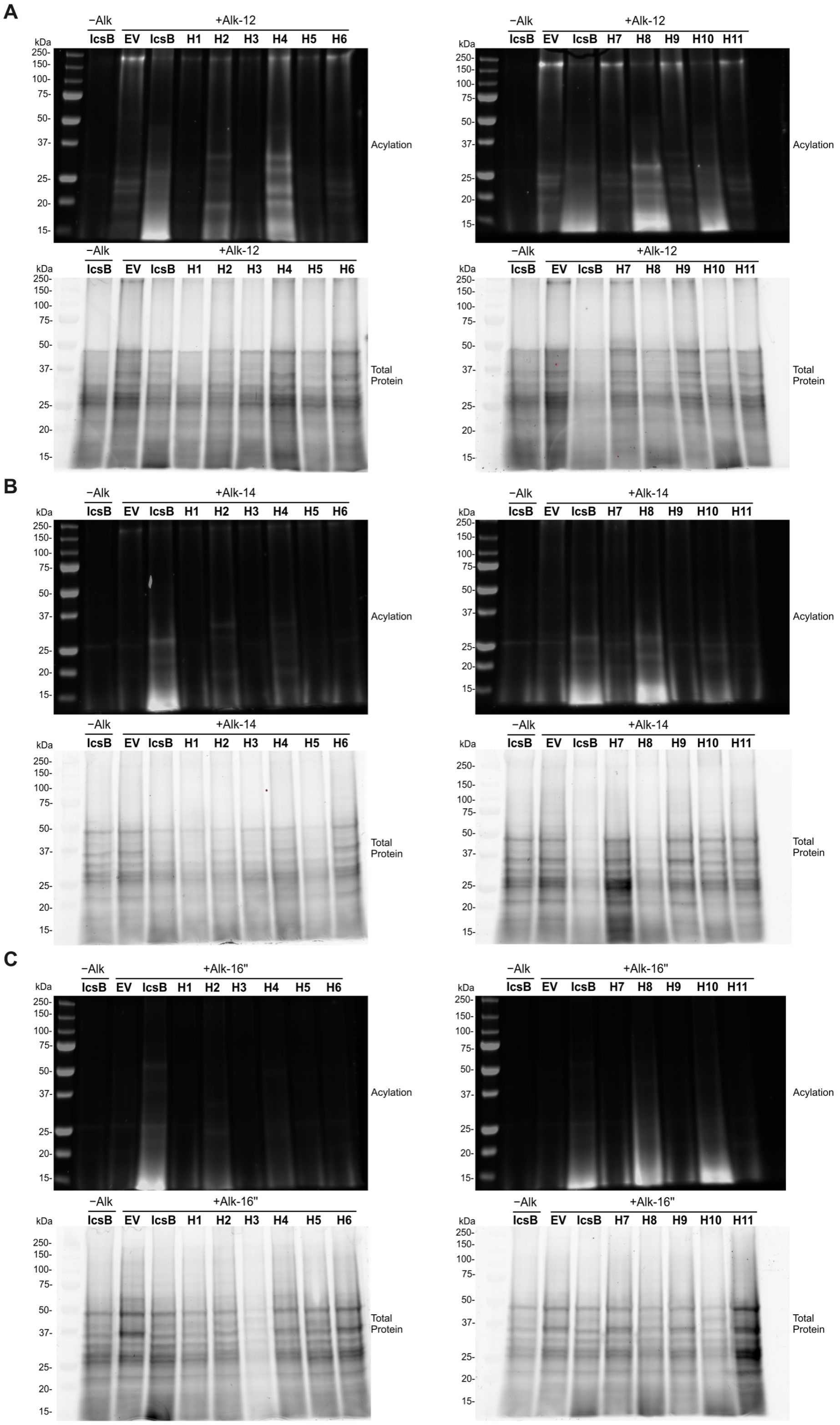

Figure S3. In-gel fluorescence using alternative fatty acid alkyne derivatives reveals the broad lipid specificity of k-FATs. In gel acylation assay obtained with (A) myristic acid (12:0, Alk-12), (B) palmitic acid (14:0, Alk-14) and (C) oleic acid (16:1, Alk-16") alkyne. The total protein in these samples were assessed using the TGX stain (bottom gel in each panel).

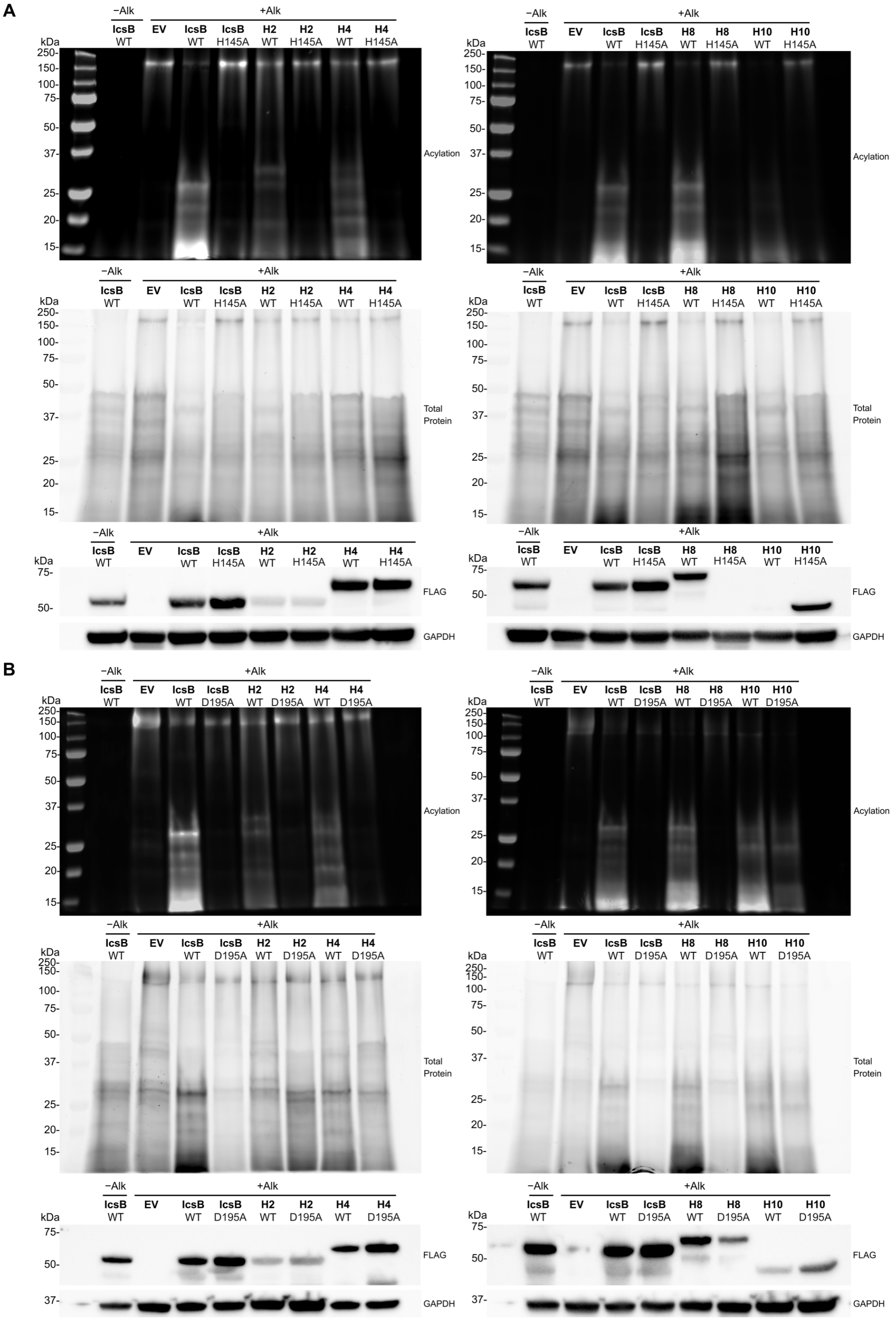

Figure S4. Characterization of the in-gel activity of H145A and D195A mutants of toxic k-FATs. (A) In-gel protein acylation of H145A mutants compared to their WT counterparts. (B) In-gel protein acylation of D195A mutants compared to their WT counterparts. Each panel shows the activity of the in-gel acylation assay (top), the total protein was assessed with the TGX stain (middle), and the expression of the acyltransferases was detected by immunoblotting with their 3× FLAG tag along with the GAPDH as a loading control (bottom).
